# Integrated multi-omics and single-cell transcriptomic analysis reveals shared molecular mechanisms and cell–cell communication signatures in gout and metabolic syndrome

**DOI:** 10.3389/fmed.2026.1749788

**Published:** 2026-04-15

**Authors:** Zichen Shao, Qinqin Deng, Ling Cheng, Fo Yang

**Affiliations:** 1 Jiangxi University of Chinese Medicine, Nanchang, Jiangxi, China; 2 Hongdu Hospital of Traditional Chinese Medicine Affiliated to Jiangxi University of Chinese Medicine, Nanchang, Jiangxi, China

**Keywords:** cell–cell communication, CSF1R, gout, JAK1, metabolic syndrome, multi-omics analysis, NAMPT, single-cell transcriptomics

## Abstract

**Objectives:**

This study aimed to comprehensively investigate the shared molecular mechanisms and intercellular communication signatures of gout and metabolic syndrome (MetS), seeking to identify and validate key regulatory genes and pathways for developing precise diagnostic and therapeutic strategies.

**Methods:**

Transcriptomic datasets for gout and MetS were retrieved from the Gene Expression Omnibus (GEO) database. Common differentially expressed genes (CODEGs) were identified through integrative analysis, followed by the construction of protein–protein interaction (PPI), drug–gene, and competing endogenous RNA (ceRNA) networks to pinpoint hub genes and regulatory axes. Single-cell RNA sequencing data were analyzed to map hub gene expression and cell–cell communication patterns. Crucially, key bioinformatic predictions were validated in established *in vitro* cell models of gout and MetS using quantitative real-time PCR (qPCR) and Western blot analysis.

**Results:**

A total of 261 CODEGs were identified, leading to the selection of 19 hub genes, including JAK1 and CSF1R. Functional enrichment analysis revealed their primary involvement in immune activation and inflammatory signaling, such as the JAK–STAT pathway. Experimental validation confirmed these findings: qPCR analysis demonstrated that the mRNA levels of JAK1, CSF1R, and NAMPT were significantly elevated in cellular models simulating both gout and MetS conditions. Furthermore, Western blot analysis revealed increased protein expression of JAK1 and CSF1R, alongside a marked increase in phosphorylated STAT3 (p-STAT3), indicating activation of the JAK–STAT pathway at the signaling level in both conditions. Single-cell analysis showed that JAK1 and CSF1R were predominantly expressed in natural killer (NK) cells and monocytes, respectively. Cell communication analysis highlighted monocytes and neutrophils as central hubs in gout, while smooth muscle cells and hematopoietic stem cells were dominant in MetS. Notably, the VISFATIN signaling pathway was highly active in both diseases, with NAMPT-associated ligand–receptor interactions, including NAMPT–(ITGA5 + ITGB1) in gout and NAMPT–INSR in MetS.

**Conclusion:**

This study, through integrated multi-omics analysis and experimental validation, identifies and characterizes a shared molecular landscape between gout and MetS. We highlight the potential roles of JAK1 and CSF1R in a shared inflammatory context, associated with activation of the JAK–STAT pathway at the signaling level. Our findings suggest that the VISFATIN signaling axis may represent a common link and delineate NAMPT-associated communication networks. These results provide insights into the intertwined pathophysiology of gout and MetS, and may offer promising avenues for joint therapeutic interventions.

## Introduction

Gout is a metabolic disorder precipitated by dysregulated purine metabolism and impaired uric acid excretion. Clinically, it is characterized by recurrent episodes of acute arthritis, persistent hyperuricemia, and the deposition of monosodium urate crystals in joints, kidneys, and other tissues (1). Driven by global shifts in lifestyle and an aging population, the prevalence of gout has steadily risen, establishing it as a significant public health concern (2). Concurrently, metabolic syndrome (MetS) represents a cluster of clinical abnormalities—including obesity, insulin resistance, hypertension, hyperglycemia, hypertriglyceridemia, and low high-density lipoprotein cholesterol—that substantially elevate the risk for cardiovascular disease and type 2 diabetes mellitus (3). A strong epidemiological link between gout and MetS has been firmly established, with both conditions sharing multiple risk factors such as obesity, insulin resistance, and chronic low-grade inflammation, suggesting a common pathophysiological foundation (4).

Previous research has illuminated the critical roles of inflammatory responses, immune dysregulation, and metabolic disturbances in the pathogenesis of both gout and MetS (5). However, the majority of existing studies have focused on a single disease or a limited biological dimension, lacking a systematic and integrative exploration of their shared pathogenic mechanisms (6). In particular, comprehensive investigations into the core genes, key signaling pathways, and intercellular communication patterns common to both conditions remain insufficient (7). Moreover, conventional bulk omics studies often overlook the crucial influence of the cellular microenvironment and intercellular interactions in shaping complex disease networks, thereby limiting the depth and breadth of mechanistic insights (8). Consequently, there is an urgent need to employ integrative multi-omics approaches combined with single-cell sequencing to uncover the shared molecular blueprint of gout and MetS and to identify novel biomarkers and therapeutic targets.

Recent advancements in multi-omics integration and single-cell RNA sequencing (scRNA-seq) technologies have opened new avenues for deciphering the mechanisms of complex diseases. By integrating transcriptomic data, protein–protein interaction (PPI) networks, competing endogenous RNA (ceRNA) regulatory networks, and drug–gene interactions, it is possible to construct a multi-dimensional and multi-layered understanding of disease pathogenesis (9). Furthermore, analyzing the single-cell-level communication landscape, particularly through ligand–receptor-based interactions, enables the dissection of dynamic exchanges among distinct cell types within the disease microenvironment. This provides crucial insights into cellular heterogeneity and pathological progression, thereby informing the design of precise therapeutic interventions (10).

In this context, the present study was designed to: (1) integrate publicly available transcriptomic datasets for gout and MetS from the GEO database to identify common differentially expressed genes (CODEGs) and pinpoint hub genes through PPI network analysis; (2) perform GO and KEGG enrichment analyses to reveal shared biological processes and signaling pathways, and construct drug–gene and ceRNA networks to explore potential regulatory relationships and therapeutic targets; (3) analyze cellular heterogeneity and gene expression patterns in gout and MetS using scRNA-seq data to define major cell types and their specific molecular signatures; and (4) utilize CellChat to systematically investigate and compare the intercellular communication networks in gout and MetS, focusing on common signaling pathways and key ligand–receptor pairs to elucidate their roles in disease progression. Ultimately, this study seeks to provide novel, systems-level insights into the shared pathogenesis of gout and MetS, identify candidate biomarkers and therapeutic targets, and lay a theoretical foundation for the development of precision medicine strategies targeting both conditions.

## Materials and methods

### Data acquisition

Microarray and high-throughput sequencing datasets associated with gout and metabolic syndrome (MetS) were retrieved from the Gene Expression Omnibus (GEO) public database. Detailed information regarding the selected datasets, including accession numbers, tissue sources, sample sizes, and experimental platforms, is summarized in Table 1. Given the inherent heterogeneity across datasets (e.g., tissue sources such as PBMCs and adipose tissue, as well as different platforms), downstream analyses were performed in a condition-specific manner to minimize potential cross-study bias.

**Table 1:** Microarray datasets related to gout and metabolic syndrome retrieved from the GEO database.

Accession number	Disease	Tissue	Number of patient samples	Number of healthy controls	Age	Sex	Experiment type	Platform
GSE160170	Gout	PBMCs	Gout patients in remission: 3 cases; gout patients during acute flare: 3 cases	6	41.0 ± 3.89	12 males	Non-coding RNA profiling by array	GPL21827
GSE98895	MetS	PBMCs	Metabolic syndrome patients: 20 cases	20	39.95 ± 13.26	–	Expression profiling by array	GPL6947
GSE211783	Gout	PBMCs	Gout patients during acute flare: 3 cases; gout patients in remission: 3 cases	0	–	–	Expression profiling by high throughput sequencing	GPL24676
GSE249089	MetS	Subcutaneous adipose tissue	Metabolic syndrome patients: 84 cases	0	–	–	Expression profiling by high throughput sequencing	GPL24676

### Differential expression analysis

Differential gene expression analysis was conducted on transcriptomic data from both gout and MetS samples using the limma R package. Following data normalization and preprocessing, a linear modeling approach combined with empirical Bayes moderation (eBayes) was applied to identify differentially expressed genes (DEGs). Raw expression data were log2-transformed when necessary, and *p*-values were adjusted using the Benjamini–Hochberg (BH) method to control the false discovery rate. Genes were considered significantly differentially expressed if they met the cutoff criteria of an absolute log2 fold change (|logFC|) >0.585 and an adjusted *p*-value (adj. *p*-value) <0.05. DEGs were identified independently within each dataset, and common DEGs (CODEGs) were obtained by intersecting results across datasets rather than merging raw expression matrices, in order to minimize potential batch effects and cross-platform bias. The resulting DEGs were visualized using volcano plots and heatmaps, with the latter displaying the top 100 DEGs ranked by absolute logFC values for enhanced clarity.

### Identification of common hub genes and PPI network construction

Common DEGs (CODEGs) between gout and MetS were identified by intersecting the respective DEG lists using a Venn diagram. These CODEGs were then input into the STRING database (version 11.5) to construct a human protein–protein interaction (PPI) network, with a minimum required interaction score set to a medium confidence level of 0.4. Only experimentally validated and database-derived interactions were retained, and isolated nodes were excluded from the network. The network topology was analyzed using the CytoNCA plugin in Cytoscape. Hub genes (HubGenes) were identified through a two-step screening process based on multiple centrality metrics, including degree centrality (DC), betweenness centrality (BC), closeness centrality (CC), eigenvector centrality (EC), local average connectivity (LAC), and network centrality (NC). The diagnostic performance of the identified HubGenes was evaluated by generating receiver operating characteristic (ROC) curves and calculating the area under the curve (AUC) for both disease conditions. ROC analysis was performed using the pROC R package.

### Functional and pathway enrichment analysis

To elucidate the biological functions of the HubGenes, Gene Ontology (GO) and Kyoto Encyclopedia of Genes and Genomes (KEGG) enrichment analyses were performed using the clusterProfiler R package. GO analysis covered three domains: biological process (BP), cellular component (CC), and molecular function (MF). Terms and pathways with a *p*-value <0.05 and a *q*-value <1 were considered significantly enriched. Results were visualized using bar plots and bubble plots.

### Construction of regulatory and interaction networks

The co-expression relationships of the HubGenes were investigated using the GeneMANIA database. A drug–gene interaction network was constructed by querying the Drug–Gene Interaction Database (DGIdb) to identify existing drugs that target the HubGenes. To explore post-transcriptional regulation, a competing endogenous RNA (ceRNA) network was built. MiRNAs targeting the HubGenes were predicted using the miRanda, miRDB, and TargetScan databases. Subsequently, lncRNAs interacting with these miRNAs were predicted using the starBase database. The resulting lncRNA–miRNA–mRNA interactions were integrated and visualized as a ceRNA network using Cytoscape.

### Preprocessing and normalization of single-cell RNA sequencing data

Publicly available single-cell RNA sequencing (scRNA-seq) data for gout and MetS were processed using the Seurat R package (version 4). Raw data were filtered to retain cells expressing at least 50 genes and having a mitochondrial gene content below 5%. Data were normalized using the “LogNormalize” method with a scale factor of 10,000, and the top 1,500 highly variable genes were identified for downstream analysis. Highly variable genes were identified using the “vst” method in the Find Variable Features function. The data were further scaled using the Scale Data function before dimensionality reduction.

Principal component analysis (PCA) was performed on the scaled data, and the top 20 statistically significant principal components (PCs), as determined by JackStraw analysis, were used for clustering. Cell neighborhood graphs were constructed using the Find Neighbors function with dims = 1:20. Cell clusters were identified using the Find Clusters function with a resolution of 0.5. Dimensionality reduction and visualization were performed using t-distributed stochastic neighbor embedding (t-SNE) for gout samples and uniform manifold approximation and projection (UMAP) for MetS samples. Cluster marker genes were identified using the FindAllMarkers function with screening criteria of |logFC| >1 and *p* < 0.05, and the top 10 marker genes for each cluster were visualized in heatmaps. Cell types were annotated using the SingleR package with the Human Primary Cell Atlas Data reference. Cell-type-specific differential expression analysis was also performed using the same cutoff criteria (|logFC| >1 and *p* < 0.05). The expression patterns of HubGenes across different cell types were visualized using violin plots, feature plots, and dot plots.

### Cell–cell communication analysis

Intercellular communication networks were inferred using the CellChat R package. A CellChat object was created based on the normalized scRNA-seq data and the CellChatDB.human ligand–receptor interaction database. A CellChat object was created using the normalized scRNA-seq data and a curated human ligand–receptor interaction database. Communication probabilities were calculated, and significant signaling pathways were identified. Cell groups with fewer than 10 cells were excluded, and interactions with *p* < 0.05 were considered significant. The analysis focused on identifying central communicative cell types, key ligand–receptor pairs, and shared signaling pathways between gout and MetS, with a particular emphasis on the VISFATIN pathway and its components.

### Cell culture and *in vitro* modeling

The human monocytic cell line THP-1 was obtained from the American Type Culture Collection (ATCC, United States). Cells were cultured in RPMI-1640 medium (Gibco, United States) supplemented with 10% fetal bovine serum (FBS, Gibco) and 1% penicillin–streptomycin at 37 °C in a 5% CO_2_ incubator. To induce differentiation into macrophage-like cells, THP-1 cells were seeded in 6-well plates at a density of 1 × 10^6^ cells/well and treated with 100 ng/mL phorbol-12-myristate-13-acetate (PMA, Sigma-Aldrich, United States) for 48 h.

After differentiation, the cells were washed with PBS and cultured in fresh serum-free medium for 12 h before stimulation. The cells were then divided into three groups:

Control Group: Cells were treated with vehicle (serum-free medium).

Gout Model Group: Cells were stimulated with 200 μg/mL of monosodium urate (MSU) crystals (InvivoGen, United States) for 6 h to mimic the acute inflammatory response of gout.

MetS Model Group: Cells were treated with a combination of high glucose (30 mM D-glucose, Sigma-Aldrich) and 200 μM palmitate (PA, Sigma-Aldrich) for 24 h to simulate the metabolic stress and low-grade inflammation characteristic of MetS.

After stimulation, cells were harvested for subsequent RNA and protein extraction.

### Quantitative real-time PCR (qPCR)

Total RNA was extracted from treated THP-1 macrophages using TRIzol reagent (Invitrogen, United States) according to the manufacturer’s protocol. cDNA was synthesized using the PrimeScript™ RT Reagent Kit (Takara, Japan). qPCR was performed on a CFX96 Real-Time PCR Detection System (Bio-Rad, United States) using SYBR Green Master Mix (Takara, Japan). The relative mRNA expression of JAK1, CSF1R, and NAMPT was calculated using the 2-ΔΔCt method and normalized to the expression of the housekeeping gene GAPDH. Primer sequences are available upon request.

### Western blot analysis

Total protein was extracted from treated THP-1 macrophages using RIPA lysis buffer (Beyotime, China) containing a protease and phosphatase inhibitor cocktail. Protein concentrations were determined using a BCA protein assay kit (Beyotime, China). Equal amounts of protein (30 μg) per sample were separated by 10% SDS-PAGE and transferred onto polyvinylidene fluoride (PVDF) membranes. The membranes were blocked with 5% non-fat milk and incubated overnight at 4 °C with primary antibodies against JAK1 (Abcam), CSF1R (Abcam), phospho-STAT3 (Tyr705) (Cell Signaling Technology), and *β*-actin (Cell Signaling Technology). After incubation with HRP-conjugated secondary antibodies, protein bands were visualized using an enhanced chemiluminescence (ECL) detection system. Band intensities were quantified using ImageJ software and normalized to *β*-actin.

## Results

### Identification of common differentially expressed genes in gout and MetS

**Figure 1:**
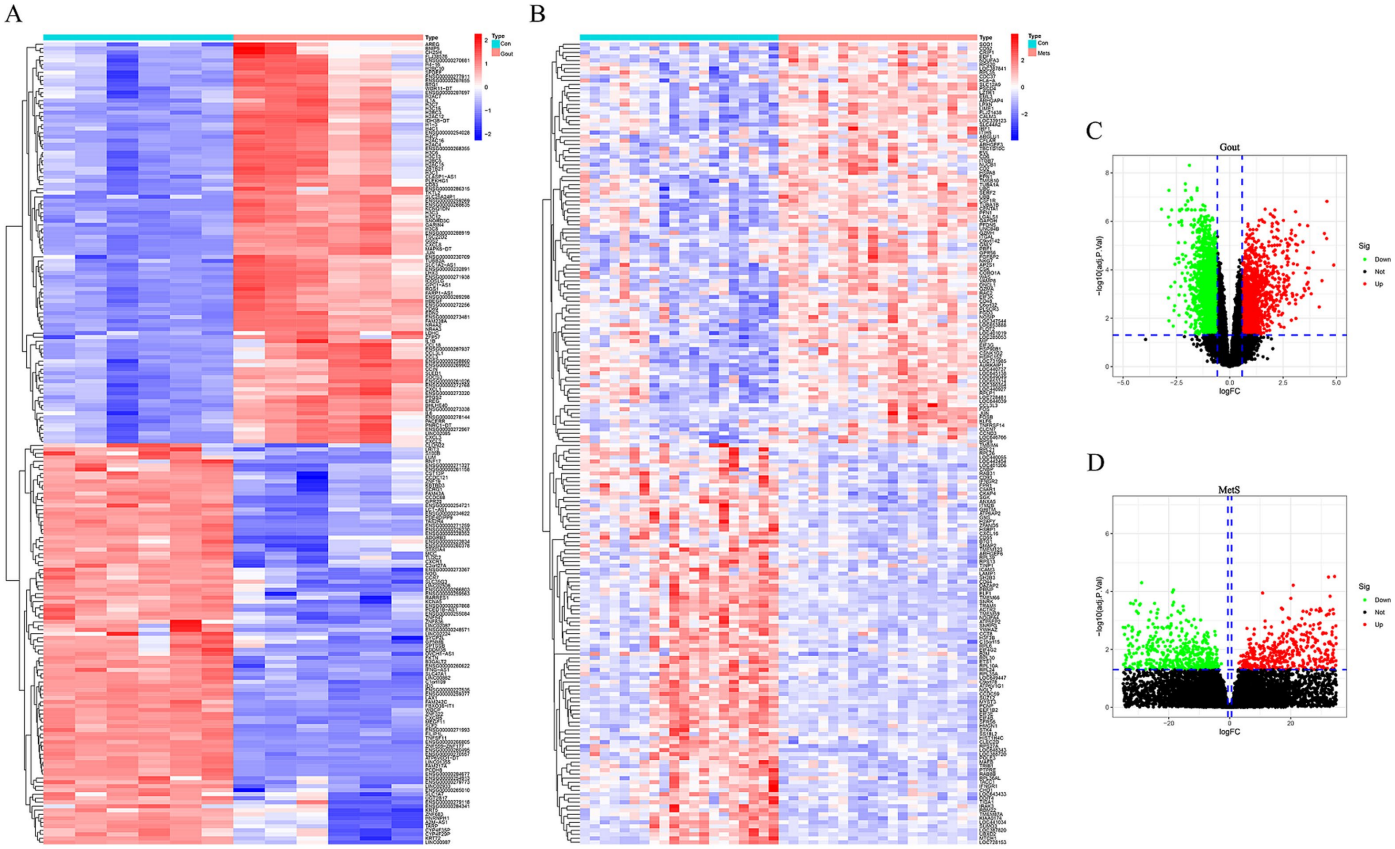
Identification of differentially expressed genes (DEGs) in gout and metabolic syndrome (MetS). **(A)** Heatmap displaying the expression of the top 100 DEGs in gout samples. **(B)** Heatmap of the top 100 DEGs in MetS samples. Red indicates upregulation and blue indicates downregulation. **(C)** Volcano plot illustrating DEGs in the gout dataset. **(D)** Volcano plot of DEGs in the MetS dataset. Red dots represent upregulated genes, and green dots represent downregulated genes meeting the criteria of |logFC| >0.585 and adj. *p*-value <0.05.

Differential expression analysis of the transcriptomic datasets identified 4,320 differentially expressed genes (DEGs) in the gout group (1,617 upregulated and 2,703 downregulated) and 2,342 DEGs in the MetS group (1,179 upregulated and 1,163 downregulated). The expression profiles of these DEGs were visualized using heatmaps and volcano plots, highlighting distinct transcriptional signatures for each condition (Figure 1).

### PPI network construction and hub gene identification

**Figure 2:**
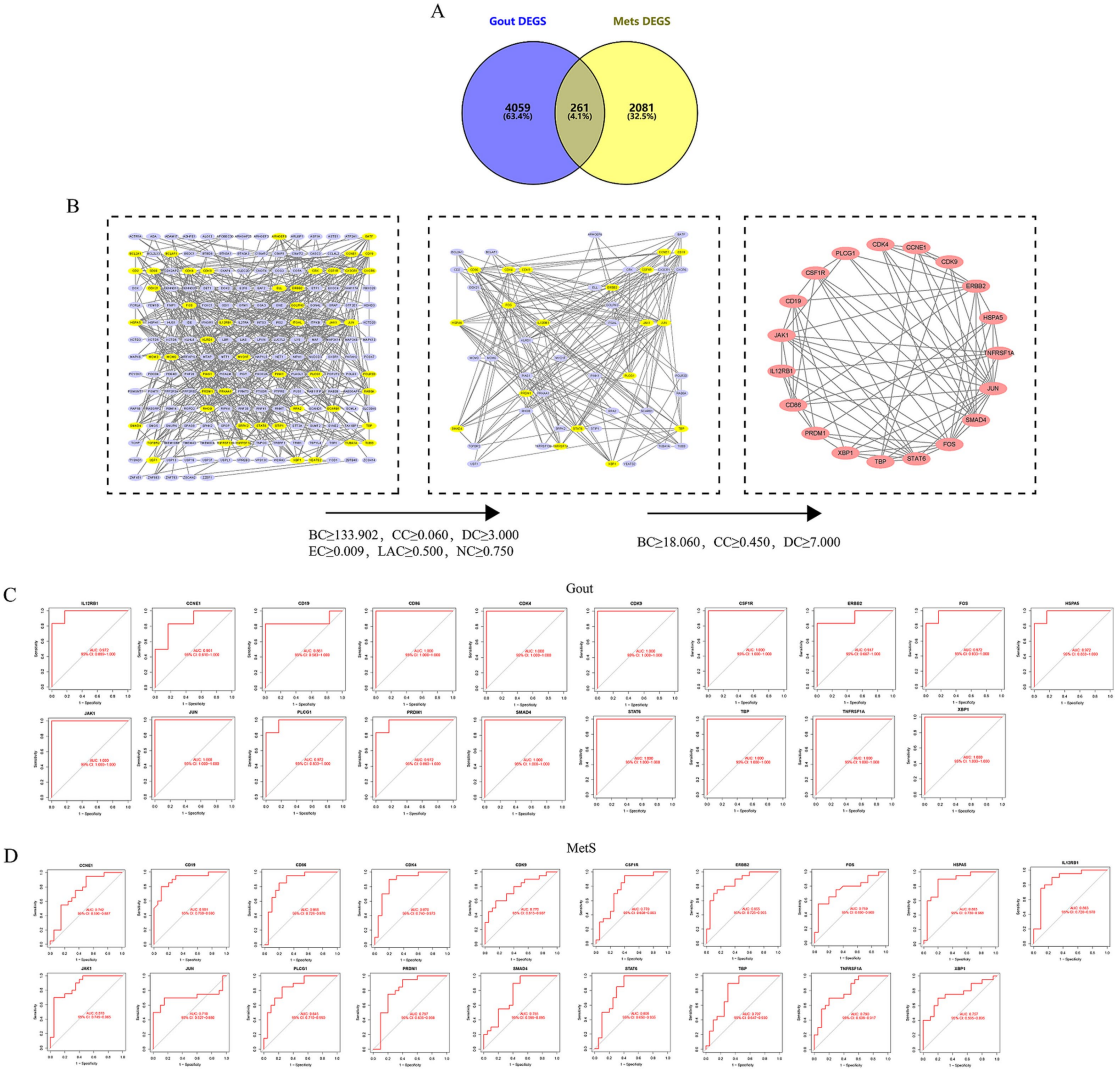
Identification of common hub genes and evaluation of their diagnostic value. **(A)** Venn diagram showing the 261 common DEGs (CODEGs) overlapping between the gout and MetS groups. **(B)** Protein–protein interaction (PPI) network of the 19 identified hub genes, constructed using STRING and refined with Cytoscape. Node size and color correspond to degree centrality. **(C)** Receiver operating characteristic (ROC) curves assessing the diagnostic performance of the 19 hub genes in the gout cohort. **(D)** ROC curves for the hub genes in the MetS cohort.

By intersecting the DEGs from both conditions, a total of 261 common DEGs (CODEGs) were identified (Figure 2A). These CODEGs were used to construct a protein–protein interaction (PPI) network in the STRING database, resulting in a network of 201 nodes and 475 edges. Topological analysis using the CytoNCA plugin in Cytoscape, performed through a two-step filtering process based on multiple centrality metrics, yielded a final core subnetwork consisting of 19 nodes and 75 edges (Figure 2B). Specifically, in the first step, nodes satisfying the criteria of BC ≥ 133.902, CC ≥ 0.060, DC ≥ 3.000, EC ≥ 0.009, LAC ≥ 0.500, and NC ≥ 0.750 were retained, resulting in a subnetwork of 51 nodes and 201 edges. In the second step, more stringent thresholds (BC ≥ 18.060, CC ≥ 0.450, and DC ≥ 7.000) were applied to identify the most interconnected nodes, yielding the final set of 19 HubGenes. This stepwise multi-parameter filtering strategy was used to enhance the robustness of hub gene selection. These 19 nodes were designated as hub genes (HubGenes) for subsequent analysis. The identified HubGenes were: JUN, CCNE1, JAK1, TBP, XBP1, SMAD4, PLCG1, TNFRSF1A, ERBB2, HSPA5, CSF1R, CD19, CDK4, IL12RB1, CD86, STAT6, PRDM1, CDK9, and FOS. To evaluate their clinical relevance, receiver operating characteristic (ROC) curve analysis was performed. All 19 HubGenes demonstrated strong diagnostic potential, with area under the curve (AUC) values exceeding 0.71 for both gout and MetS, indicating their robustness as potential biomarkers (Figures 2C,D).

### Functional and pathway enrichment of HubGenes

**Figure 3:**
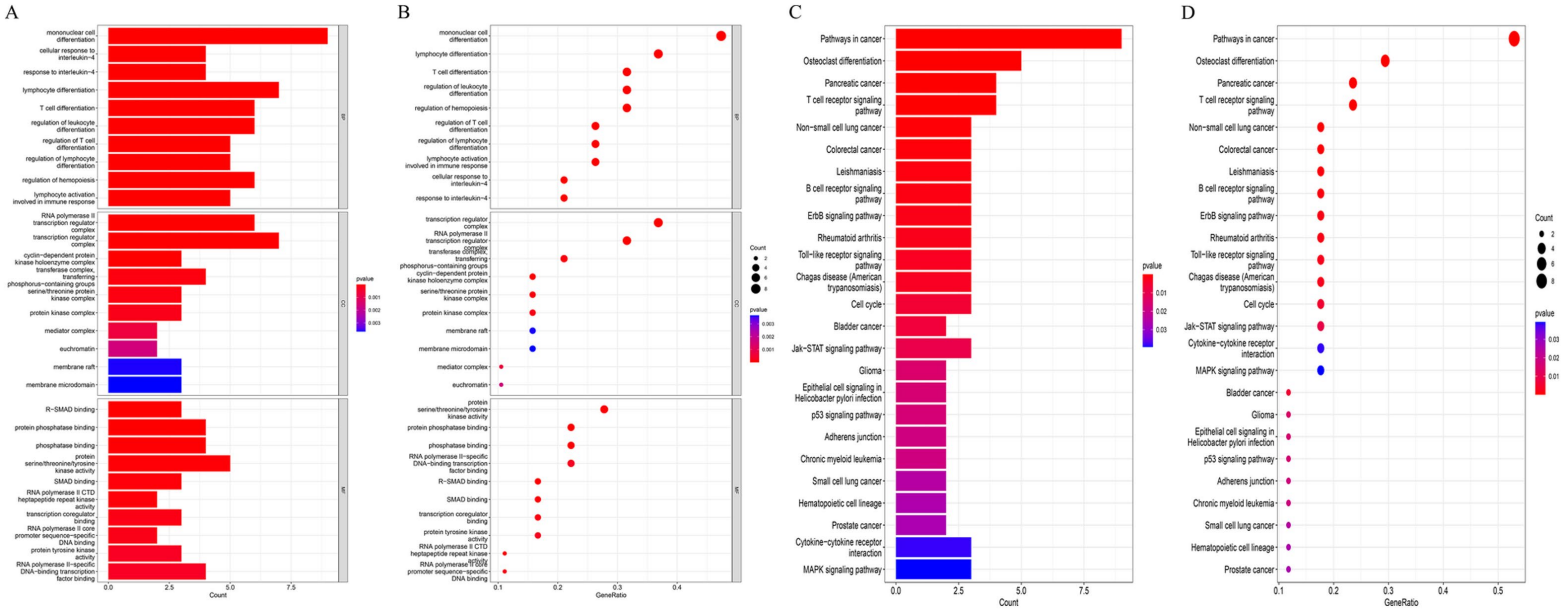
Functional enrichment analysis of the 19 hub genes. **(A)** Bar plot and **(B)** bubble plot visualizing the top enriched Gene Ontology (GO) terms in the categories of biological process (BP), cellular component (CC), and molecular function (MF). **(C)** Bar plot and **(D)** bubble plot showing the top enriched Kyoto Encyclopedia of Genes and Genomes (KEGG) pathways. Dot size represents gene count, and color indicates statistical significance.

To elucidate the biological roles of the 19 HubGenes, GO and KEGG enrichment analyses were performed. GO analysis revealed significant enrichment in processes related to immune cell regulation, including the differentiation and activation of monocytes, lymphocytes, and T cells, as well as responses to cytokines like interleukin-4 (Figures 3A,B). KEGG pathway analysis highlighted their involvement in critical signaling cascades, such as the T cell receptor, B cell receptor, ErbB, Toll-like receptor, JAK–STAT, and MAPK pathways. Additionally, these genes were associated with cellular processes like osteoclast differentiation and the cell cycle, and were implicated in various diseases, including several types of cancer and autoimmune disorders (Figures 3C,D).

### Construction of co-expression, drug–gene, and ceRNA networks

**Figure 4:**
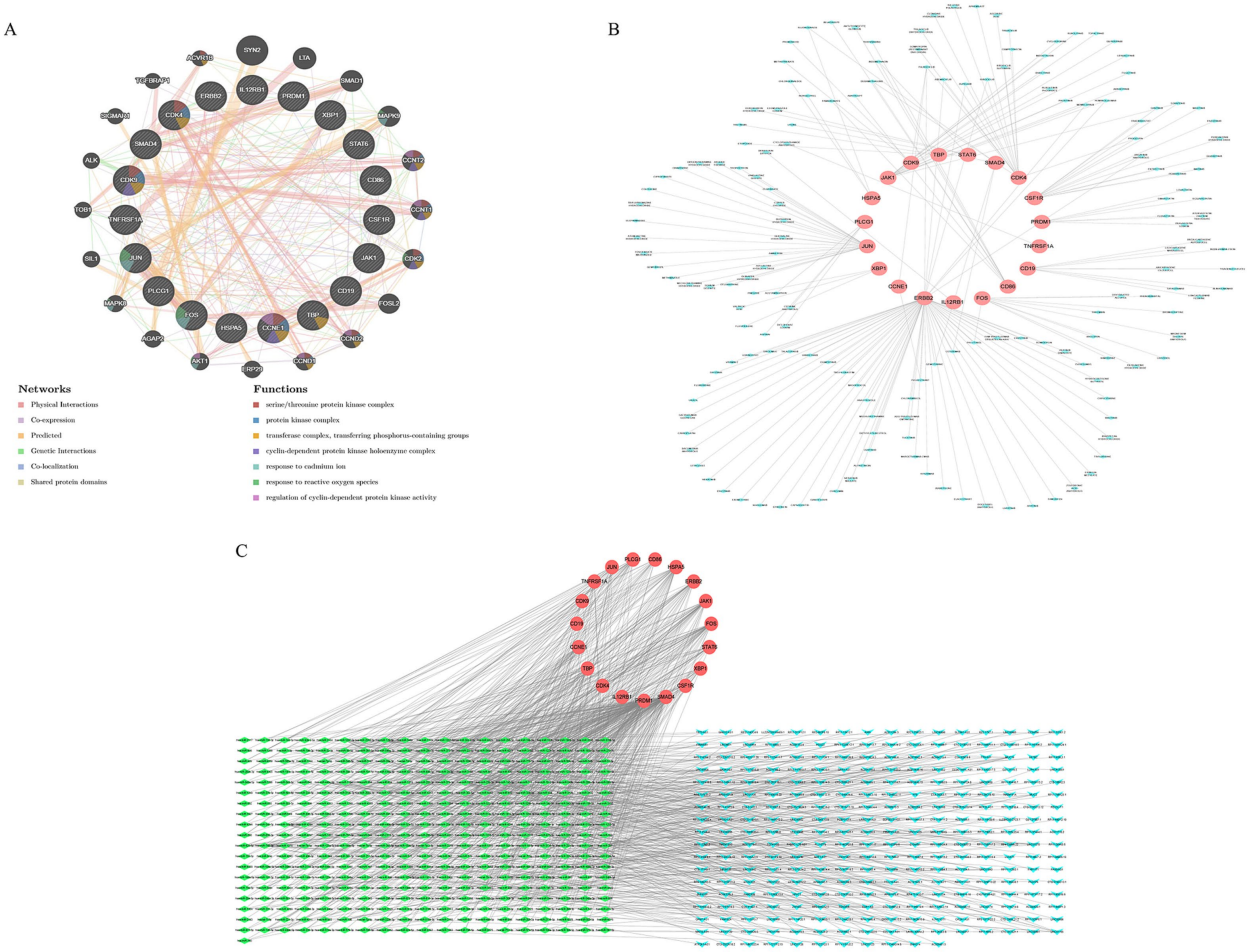
Construction of regulatory and interaction networks for the hub genes. **(A)** Co-expression network of the 19 hub genes generated by GeneMANIA, illustrating various types of interactions (physical, co-expression, genetic, etc.). **(B)** Drug-gene interaction network showing 166 candidate drugs targeting the hub genes. **(C)** The competing endogenous RNA (ceRNA) regulatory network, depicting interactions between lncRNAs, miRNAs, and the 19 hub gene mRNAs.

The regulatory landscape of the HubGenes was further explored by constructing multiple interaction networks. A co-expression network generated using GeneMANIA revealed a complex web of interactions, including physical interactions (40.22%), co-expression (24.93%), and predicted functional relationships (22.96%) (Figure 4A). A drug–gene interaction network, built using the DGIdb database, identified 166 existing drugs targeting the HubGenes, with ERBB2, JUN, and CDK4 being the most frequently targeted genes (Figure 4B). Finally, a competing endogenous RNA (ceRNA) network was constructed, comprising 283 lncRNAs, 362 miRNAs, and the 19 HubGene mRNAs, connected by 966 regulatory edges. This network provides a comprehensive view of the post-transcriptional regulatory mechanisms governing HubGene expression (Figure 4C).

### Single-cell transcriptomic profiling of gout and MetS

**Figure 5:**
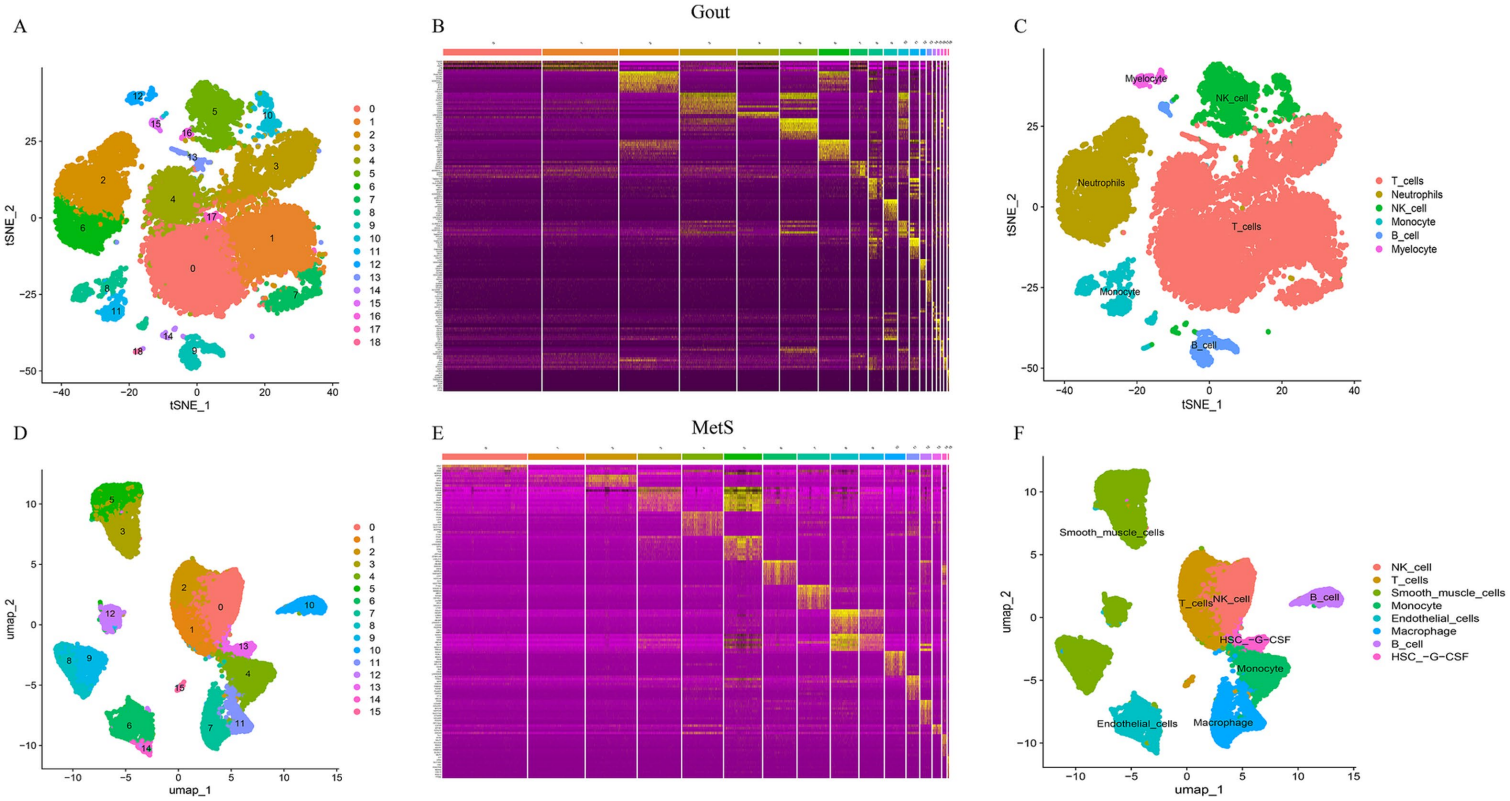
Cell clustering and annotation in gout and MetS. **(A)** *t*-SNE plot showing the 19 identified cell clusters in gout samples. **(B)** Heatmap of the top 10 marker genes for each cluster in gout. **(C)** Cell type annotation of the gout clusters. **(D)** UMAP plot of the 16 cell clusters in MetS samples. **(E)** Heatmap of top 10 marker genes in MetS. **(F)** Cell type annotation of the MetS clusters.

Following data preprocessing and quality control, principal component analysis confirmed significant cellular heterogeneity in both gout and MetS samples. Subsequent clustering analysis identified 19 distinct cell clusters in gout and 16 in MetS. Cell type annotation using the SingleR package revealed that the gout microenvironment was dominated by immune cells such as T cells, neutrophils, NK cells, and monocytes. In contrast, the MetS microenvironment was characterized by NK cells, T cells, smooth muscle cells, monocytes, and macrophages (Figure 5).

**Figure 6:**
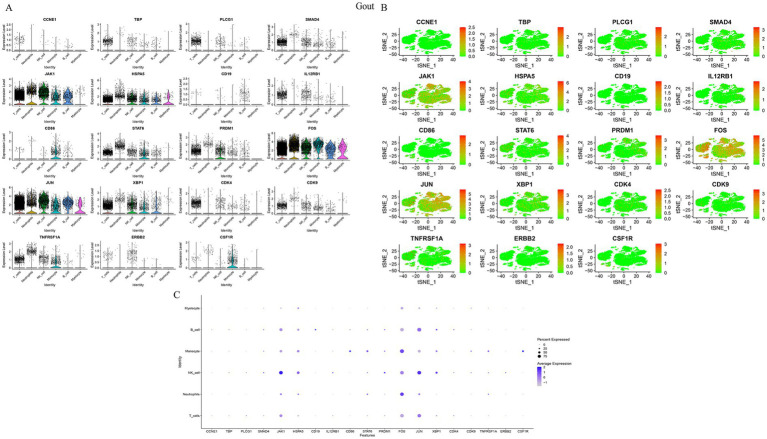
Visualization of hub gene expression in gout samples. **(A)** Violin plots showing the expression distribution of key hub genes across different cell types. **(B)** Feature plots (*t*-SNE) illustrating the spatial expression patterns of selected hub genes. **(C)** Dot plot summarizing the expression level and percentage of expressing cells for hub genes across all cell types.

**Figure 7:**
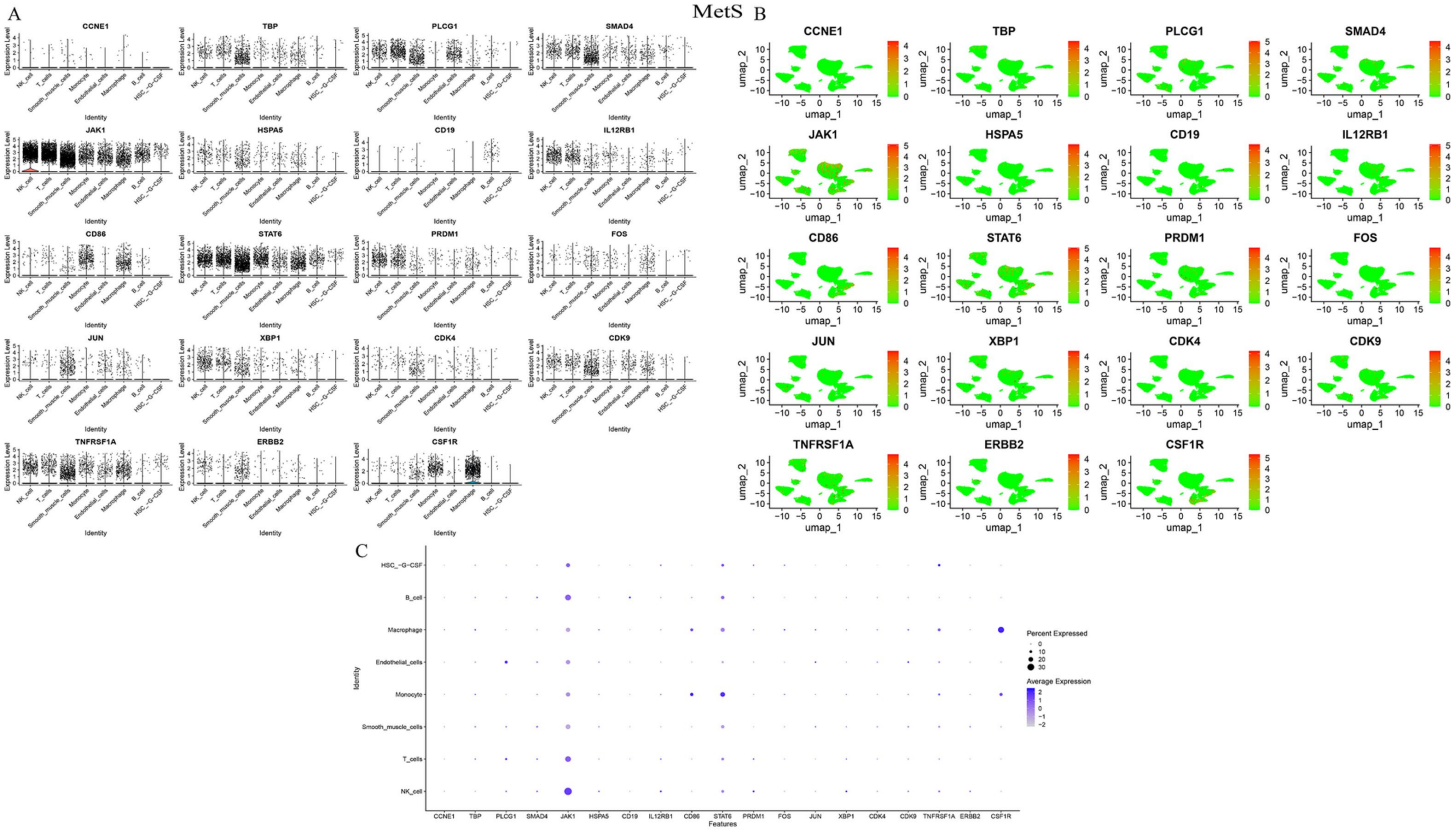
Visualization of hub gene expression in MetS samples. **(A)** Violin plots, **(B)** feature plots (UMAP), and **(C)** dot plot visualizing the expression of key hub genes across different cell types in MetS.

Visualization of HubGene expression at the single-cell level revealed distinct cell-type specificities. In gout samples, JUN, XBP1, and PRDM1 were highly expressed in NK cells, while CSF1R and CD86 were enriched in monocytes. JAK1 was notably upregulated in NK cells across both diseases (Figures 6, 7). In MetS samples, JAK1 maintained high expression in NK cells, and CSF1R was predominantly found in macrophages (Figure 7). Based on their consistent upregulation and central roles, JAK1 and CSF1R were selected as key genes for further investigation (Table 2).

**Table 2:** Differential expression profiles of JAK1 and CSF1R in single-cell sequencing data from gout and MetS samples.

Samples	Gene	Cluster	logFC	Adj. *p*-value	Up/down
Gout	XBP1	NK_cell	1.269147151	1.64*E* − 127	Up
	PRDM1	NK_cell	1.246201925	9.46*E* − 123	Up
	JUN	NK_cell	1.070587822	7.80*E* − 151	Up
	JUN	Monocyte	−1.168928963	6.15*E* − 48	Down
	JUN	Myelocyte	−1.286597733	1.37*E* − 15	Down
	JAK1	NK_cell	1.215501076	4.40*E* − 272	Up
	HSPA5	T_cells	−1.752721624	1.22*E* − 27	Down
	HSPA5	Neutrophils	1.549276562	4.84*E* − 46	Up
	FOS	T_cells	−1.186439975	8.02*E* − 246	Down
	FOS	Neutrophils	1.337157708	1.14*E* − 223	Up
	CSF1R	Monocyte	4.944921806	0	Up
	CD86	Monocyte	5.380506909	0	Up
MetS	JAK1	NK_cell	1.110129495	5.45*E* − 131	Up
	CSF1R	Macrophage	3.904567191	0	Up

### Dissection of cell–cell communication networks

**Figure 8:**
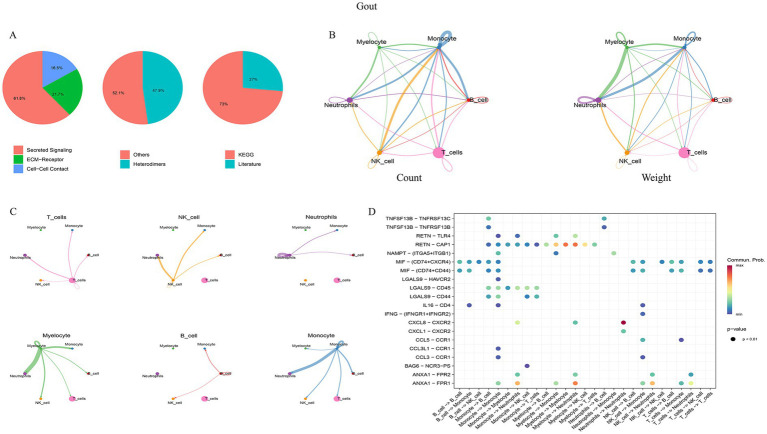
Cell–cell communication network in gout samples. **(A)** Donut plot showing the proportion of communication pathways classified as secreted signaling, ECM-receptor, and cell–cell contact. **(B)** Circle plot visualizing the overall number and strength of interactions between all identified cell types. **(C)** Network diagram highlighting the specific outgoing and incoming signals of monocytes. **(D)** Bubble plot of key ligand-receptor pairs mediating communication between major cell types.

**Figure 9:**
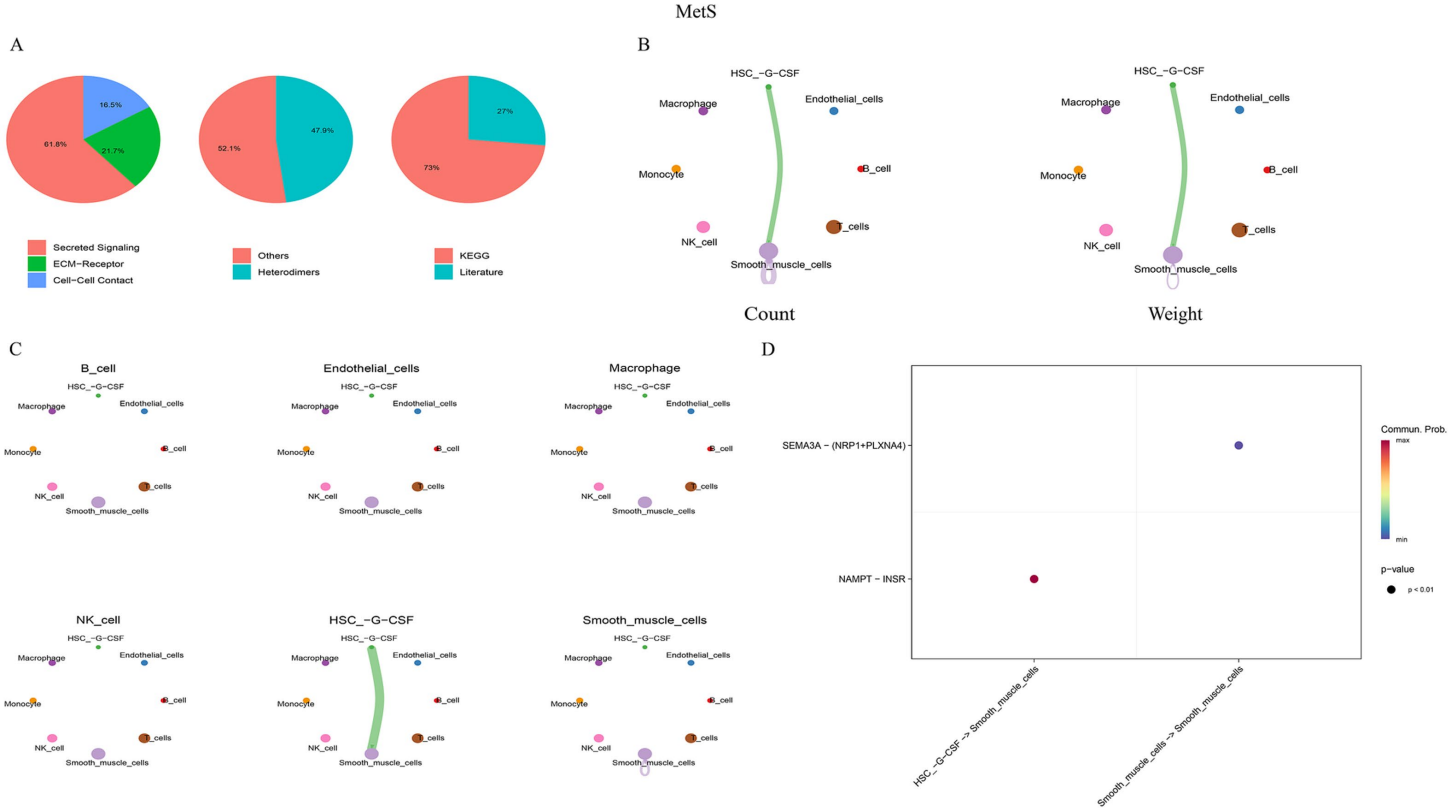
Cell–cell communication network in MetS samples. **(A)** Classification of communication pathways. **(B)** Global intercellular communication network. **(C)** HSC-specific interaction network. **(D)** Bubble plot showing key ligand-receptor interactions, particularly between HSCs and smooth muscle cells.

Cell–cell communication analysis using CellChat revealed distinct interaction patterns. In gout, monocytes and T cells were the most active communicators, with monocytes acting as a major signaling hub (Figure 8). In MetS, communication was dominated by interactions between smooth muscle cells and hematopoietic stem cells (HSCs) (Figure 9).

**Figure 10:**
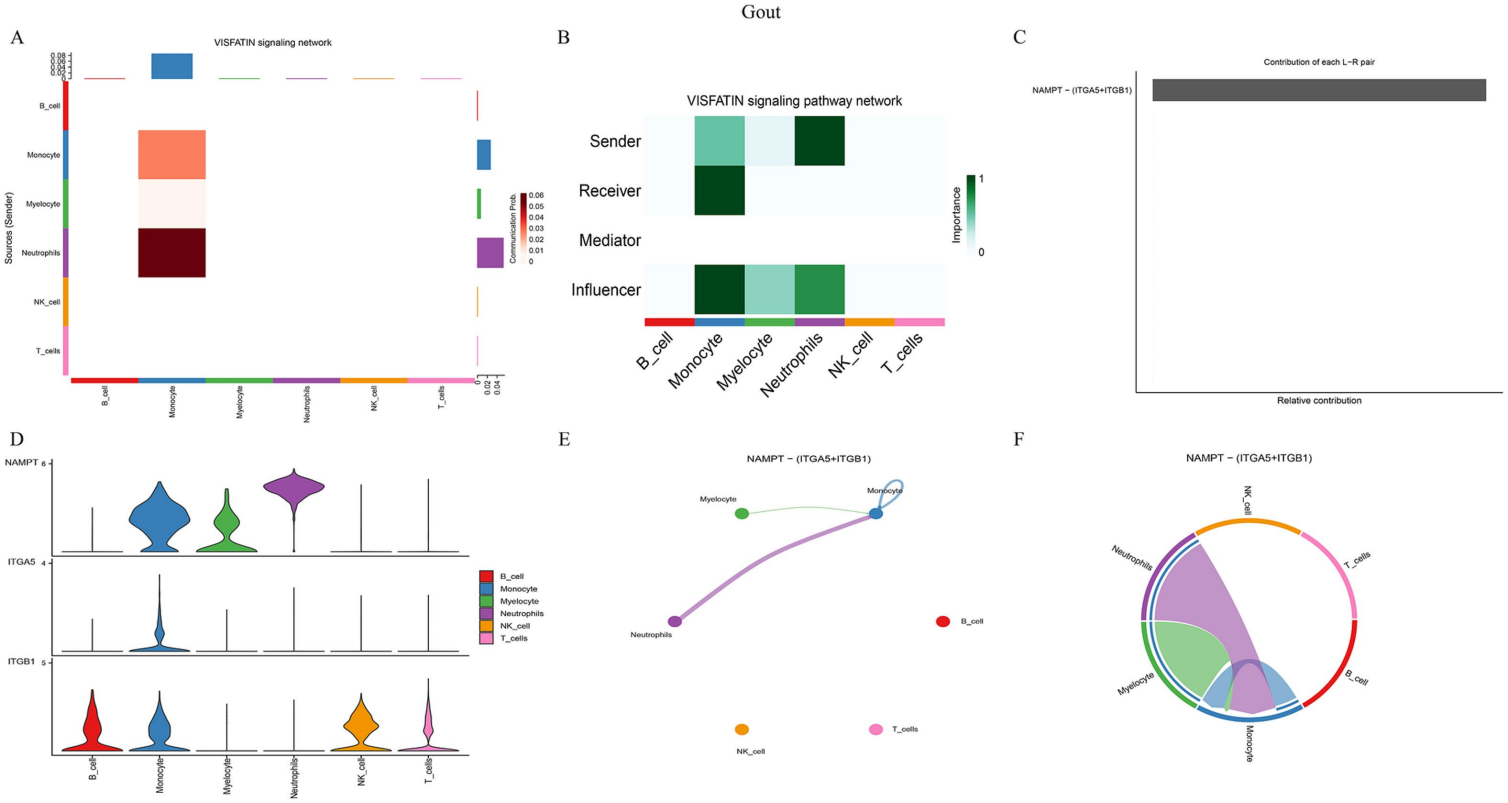
Characterization of the VISFATIN signaling pathway in gout. **(A)** Circle plot showing the communication strength of the VISFATIN pathway among monocytes, myelocytes, and neutrophils. **(B)** Role assignment of each cell type (Sender, Receiver, etc.) within the pathway. **(C)** Contribution analysis of the NAMPT–(ITGA5 + ITGB1) ligand-receptor pair to the overall signaling. **(D)** Violin plots showing the expression of NAMPT, ITGA5, and ITGB1. **(E)** Network diagram and **(F)** chord diagram illustrating the directional communication mediated by NAMPT–(ITGA5 + ITGB1).

**Figure 11:**
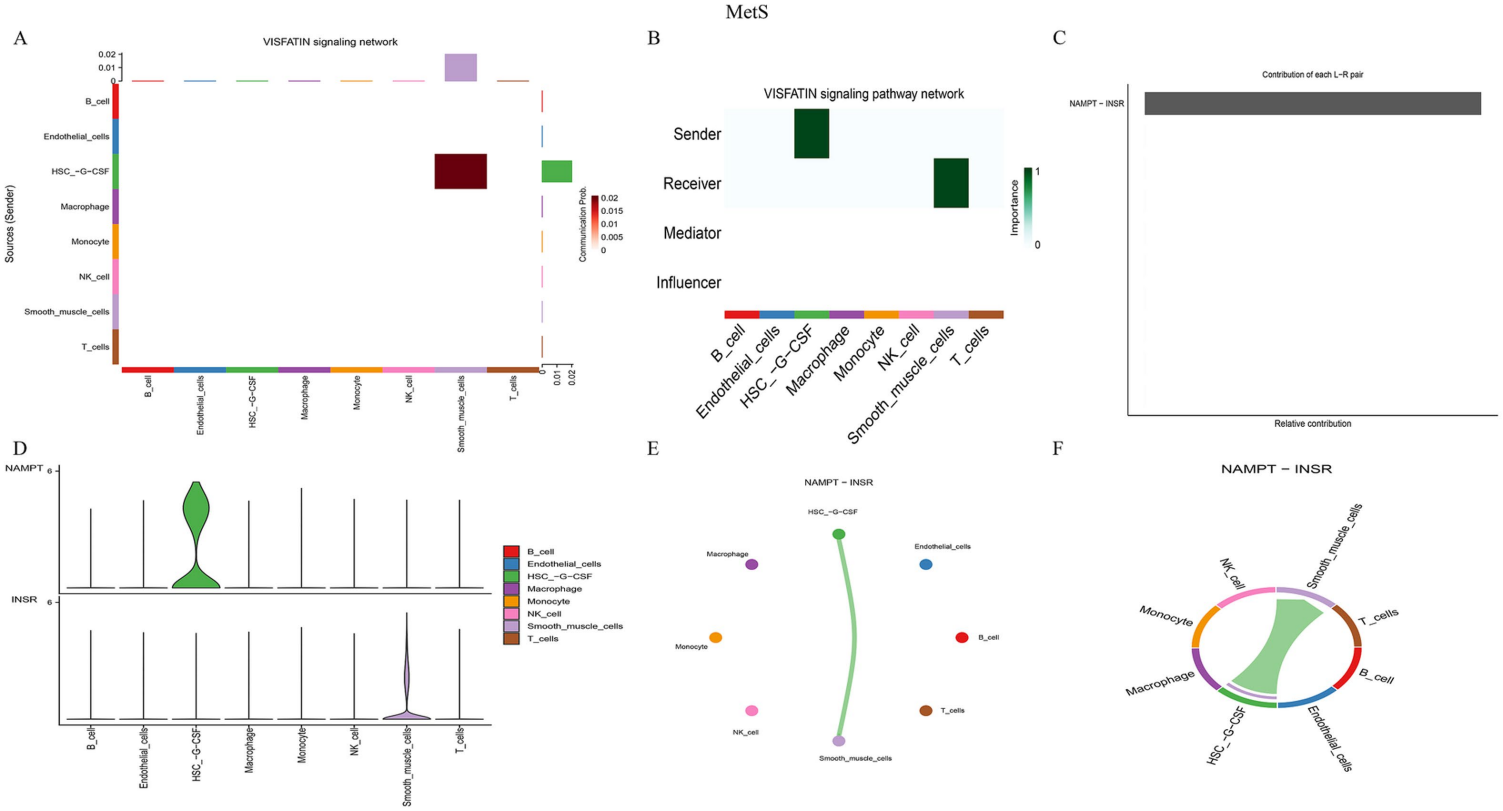
Characterization of the visfatin signaling pathway in MetS. **(A)** Communication strength between HSCs and smooth muscle cells. **(B)** Role assignment within the visfatin pathway. **(C)** Contribution analysis of the NAMPT–INSR pair. **(D)** Violin plots of NAMPT and INSR expression. **(E)** Network diagram and **(F)** chord diagram visualizing the communication mediated by NAMPT–INSR.

Notably, the VISFATIN signaling pathway was identified as a shared, highly active pathway in both conditions. In gout, this pathway was primarily driven by the NAMPT–(ITGA5 + ITGB1) ligand–receptor pair, mediating communication between monocytes and neutrophils (Figure 10). In MetS, the key interaction was NAMPT–INSR, facilitating signaling from HSCs to smooth muscle cells (Figure 11). These findings pinpoint NAMPT-mediated signaling as a critical, albeit context-specific, communication axis in both diseases.

### Experimental validation in *in vitro* models confirms upregulation of key hub genes and activation of the JAK–STAT pathway

To experimentally validate our key bioinformatic findings, we established *in vitro* models of gout and MetS using PMA-differentiated THP-1 macrophages and performed qPCR and Western blot analyses. The cells were assigned to a control group, a gout model group (stimulated with MSU crystals), and a MetS model group (stimulated with high glucose and palmitate).

**Figure 12:**
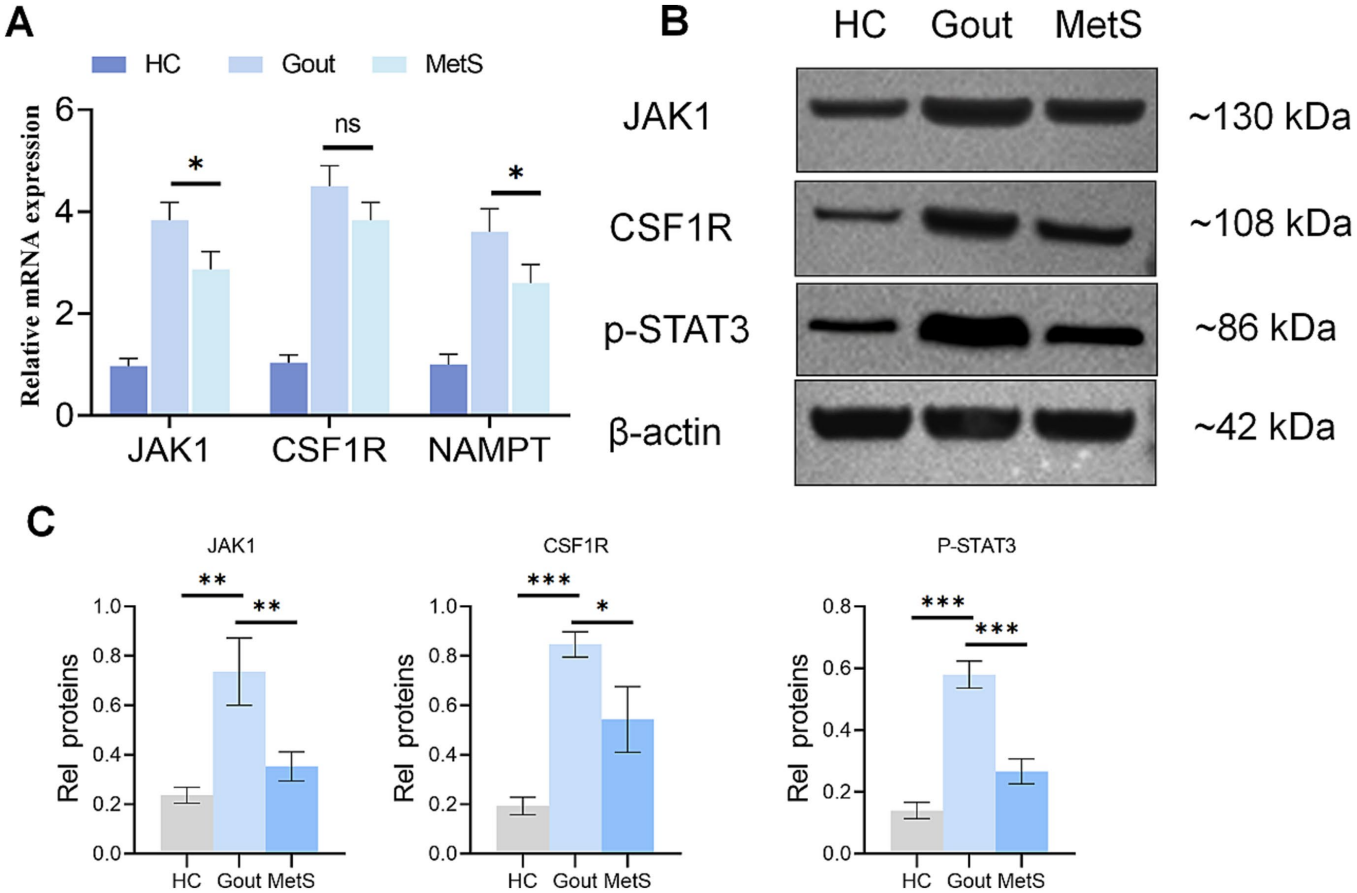
Experimental validation of key hub genes and pathways. **(A)** Relative mRNA expression of JAK1, CSF1R, and NAMPT in THP-1 macrophages under different stimulation conditions, as measured by qPCR. “Control” represents untreated cells; “Gout” represents cells stimulated with MSU crystals; “MetS” represents cells stimulated with high glucose and palmitate. **(B)** Representative western blot images showing the protein levels of JAK1, CSF1R, phosphorylated STAT3 (p-STAT3), and *β*-actin (loading control) in the three experimental groups. **(C)** Quantification of the western blot band densities, normalized to β-actin. Data are presented as mean ± SD from three independent experiments. Statistical significance was determined by one-way ANOVA (*p < 0.05, **p < 0.01, ***p < 0.001).

The qPCR results demonstrated that the relative mRNA expression of the hub genes JAK1 and CSF1R, along with the key signaling molecule NAMPT (Visfatin), was significantly upregulated in both the MSU-stimulated (gout model) and HG + PA-stimulated (MetS model) groups compared to the untreated control group (Figure 12A). This provides strong evidence at the transcriptomic level for their shared involvement in both pathologies.

To confirm these findings at the protein level and assess functional pathway activation, we conducted Western blot analysis. Consistent with the qPCR data, protein expression of JAK1 and CSF1R was markedly increased in both the gout and MetS model groups (Figures 12B,C). Crucially, we observed a significant increase in the phosphorylation of STAT3 (p-STAT3), a key downstream effector of the JAK1 signaling cascade. The level of p-STAT3 was minimal in control cells but substantially elevated in both stimulated disease model groups, indicating activation of the JAK–STAT signaling pathway at the phosphorylation level (Figures 12B,C).

Collectively, these experimental results provide robust validation for our computational predictions, confirming that JAK1, CSF1R, and the NAMPT-driven JAK–STAT signaling axis are indeed dysregulated and exhibit activation-associated molecular changes under conditions mimicking both gout and metabolic syndrome *in vitro*.

## Discussion

Gout and metabolic syndrome (MetS) are intrinsically linked through shared pathological features, particularly metabolic dysregulation and chronic inflammation. However, the precise molecular mechanisms underlying their comorbidity have remained incompletely understood. In this study, we conducted a comprehensive investigation by integrating bulk transcriptomic data, protein–protein interaction (PPI) networks, functional enrichment analyses, ceRNA regulatory interactions, and single-cell RNA sequencing (scRNA-seq) to systematically dissect the shared pathogenic basis of these two conditions. Our multi-omics approach identified 261 common differentially expressed genes (CODEGs), from which 19 hub genes with high diagnostic potential were prioritized. Functional analysis revealed that these genes are primarily involved in immune cell differentiation and classical inflammatory signaling pathways, including T/B cell receptor, JAK–STAT, and MAPK pathways. Single-cell transcriptomic analysis further highlighted the elevated expression of key genes like JAK1 and CSF1R within specific immune cell populations. Crucially, our experimental validation in established *in vitro* models confirmed the significant upregulation of JAK1, CSF1R, and NAMPT, alongside the functional activation of the JAK–STAT pathway. Cell–cell communication analysis pinpointed the NAMPT–(ITGA5 + ITGB1) axis in gout and the NAMPT–INSR axis in MetS as critical signaling modules, underscoring their central role in mediating both inflammatory and metabolic dysregulation.

The identified CODEGs and HubGenes form a critical molecular nexus of immune-metabolic crosstalk. Among the 19 HubGenes, JAK1 and CSF1R emerged as central nodes. JAK1, a canonical activator of the JAK–STAT pathway, is a critical mediator linking inflammation to metabolic imbalance, and JAK1 inhibitors have demonstrated therapeutic potential in alleviating insulin resistance in MetS models (11). CSF1R, predominantly expressed in the monocyte–macrophage lineage, governs immune cell proliferation and activation, and its inhibition has shown synergistic effects in improving metabolic function and suppressing chronic inflammation (12). Our study not only predicted their importance but also experimentally validated the increased expression of both JAK1 and CSF1R at both the mRNA and protein levels. More importantly, the observed increase in phosphorylated STAT3 provides direct evidence of a hyperactive JAK–STAT “inflammation–metabolic dysregulation feedback loop” common to both diseases. This validated feedback loop offers a robust molecular framework for the comorbidity of gout and MetS and suggests novel strategies for dual-targeted therapeutic intervention.

The strong diagnostic potential of the HubGenes, indicated by AUC values exceeding 0.71, was reinforced by the functional significance of JAK1 and CSF1R. JAK1 inhibitors like baricitinib have been shown to ameliorate metabolic disturbances in inflammatory models, supporting its utility as a clinical biomarker (13, 14). Similarly, CSF1R has been established as a highly accurate diagnostic marker in various immune-metabolic diseases, reflecting systemic inflammatory status (14, 15). Functional enrichment analysis further substantiated these findings, implicating the HubGenes in immune cell activation and interleukin-mediated responses, consistent with the known roles of IL-4-dependent macrophage polarization in metabolic inflammation (16). The enrichment of pathways such as JAK–STAT, MAPK, and ErbB aligns with existing literature, where these cascades are known to govern pro-inflammatory signaling, cellular stress, and metabolic reprogramming in both conditions (17–20). These results collectively support a unified pathogenic model where immune activation and metabolic reprogramming are deeply intertwined, although the initiating triggers may differ between gout (immune-driven) and MetS (metabolic-driven) (5, 21, 22).

Our constructed ceRNA network uncovered complex post-transcriptional regulatory layers, identifying axes like SNHG1–miR-137–JUN and UCA1–miR-23b-3p–FOS, which have been previously implicated in inflammatory and metabolic disorders (23, 24). The drug-gene network analysis highlighted ERBB2, JUN, and CDK4 as highly druggable targets, with existing inhibitors for these proteins showing potential immunomodulatory and metabolic effects in preclinical models (25). These findings suggest that the identified HubGenes and their regulatory networks represent promising avenues for therapeutic intervention. However, it should be noted that the ceRNA and drug–gene networks in this study are based on database-driven predictions and should be interpreted as exploratory findings that require further experimental validation.

Single-cell analysis provided unprecedented resolution, revealing distinct cellular compositions and HubGene expression patterns. The gout microenvironment was characterized by an acute inflammatory signature with enriched neutrophils and monocytes, whereas MetS displayed a chronic inflammatory profile with dominant NK cells, macrophages, and smooth muscle cells (26, 27). The cell-type-specific expression of JAK1 in NK cells and CSF1R in monocytes/macrophages across both diseases suggests that these genes drive pathological phenotypes through distinct cellular players, leading to acute inflammation in gout versus chronic, low-grade inflammation in MetS.

Furthermore, the cell–cell communication analysis elucidated the specific signaling axes driving these phenotypes. In gout, the NAMPT–(ITGA5 + ITGB1) interaction between monocytes and neutrophils likely amplifies inflammatory signals, a mechanism previously associated with arthritic conditions (28, 29). In contrast, the NAMPT–INSR axis in MetS, operating between HSCs and smooth muscle cells, likely contributes to metabolic dysfunction and inflammatory hematopoiesis by interfering with insulin signaling (29). Notably, this apparent receptor shift may reflect context-dependent signaling adaptations of NAMPT in distinct disease microenvironments (30). Integrin-mediated signaling, such as via ITGA5/ITGB1, is closely associated with immune cell adhesion, migration, and rapid inflammatory activation (31, 32), which may facilitate acute immune cell recruitment and amplification of inflammatory responses in gout. In contrast, NAMPT engagement with INSR may preferentially influence metabolic signaling pathways, contributing to insulin resistance and sustaining chronic low-grade inflammation in MetS (33). This differential receptor usage may also have implications for immune cell polarization and functional states. For example, integrin-associated NAMPT signaling may favor pro-inflammatory activation and recruitment of neutrophils and classical monocytes (31), whereas NAMPT–INSR interactions may be more closely linked to metabolic reprogramming of macrophages and the maintenance of chronic inflammatory phenotypes (34). In patients with coexisting gout and metabolic dysfunction, such receptor plasticity may contribute to a dynamic interplay between acute and chronic inflammatory responses. Importantly, our study is the first to propose the VISFATIN (NAMPT) pathway as a convergent signaling hub in both diseases. This hypothesis is now strengthened by our experimental data showing significantly elevated NAMPT mRNA levels in both our gout and MetS cellular models. Visfatin is a known pleiotropic adipokine that promotes pro-inflammatory cytokine secretion and disrupts insulin signaling, making it a perfect molecular bridge between inflammation and metabolic imbalance (35, 36). Importantly, beyond its immunometabolic role, NAMPT/visfatin may also function within an immunolipid regulatory context. In metabolic syndrome, the lipid-rich microenvironment—characterized by elevated free fatty acids, dyslipidemia, and cholesterol accumulation—can modulate NAMPT expression and secretion. Lipotoxic stimuli such as palmitate have been shown to enhance inflammatory activation in monocytes and macrophages, potentially synergizing with NAMPT-driven signaling. In this context, NAMPT may act as a mediator linking lipid-induced metabolic stress to immune activation, further amplifying pro-inflammatory responses. Moreover, altered lipid metabolism may influence NAMPT-associated signaling pathways, including interactions with integrins and insulin receptors, thereby shaping cell–cell communication networks observed in our study (37–40). The delineation of these specific NAMPT-mediated axes provides a mechanistic basis for interventions aimed at disrupting these maladaptive cellular communication networks.

This study demonstrates significant methodological innovation by creating a comprehensive multi-omics framework that bridges bioinformatics discovery with experimental validation. By integrating bulk and single-cell data and confirming key predictions in cell samples, we have enhanced the precision and biological interpretability of our findings. Theoretically, we have not only proposed but also provided initial experimental support for the central role of the VISFATIN/JAK–STAT axis in the shared pathogenesis of gout and MetS.

Despite these strengths, our study has limitations. The use of public datasets from varied sources may introduce heterogeneity. In addition, given the intrinsic heterogeneity of metabolic syndrome, subtype-specific transcriptomic differences were not explored in the present study. The experimental validation, while crucial, was conducted using a simplified *in vitro* system based on the THP-1 cell line. While this approach allows for controlled investigation of specific molecular pathways, it does not fully recapitulate the complex cellular heterogeneity and systemic interplay of the *in vivo* microenvironment in human patients. Moreover, the experimental models for gout and MetS were established separately rather than as a combined comorbidity model, and thus do not fully capture potential interactions between the two conditions. The response of a single cell line to specific stimuli (MSU crystals for gout; high glucose and palmitate for MetS) is a reductionist model of the overall pathophysiology. In addition, although increased STAT3 phosphorylation supports activation of the JAK–STAT pathway at the signaling level, it does not fully capture downstream functional events such as STAT nuclear translocation. Therefore, our findings should be interpreted as indicative of pathway activation rather than comprehensive functional validation. Moreover, machine learning-based feature selection and external cohort validation were not performed, which may limit the generalizability of the identified hub genes. Future work should incorporate spatial transcriptomics and validation in primary cells or animal models to causally link JAK1, CSF1R, and the NAMPT signaling axes to disease pathophysiology, paving the way for their clinical translation as validated biomarkers and therapeutic targets.

## Conclusion

By integrating multi-omics data with single-cell transcriptomic analysis, and supporting key bioinformatic predictions with targeted *in vitro* experiments, this study characterizes the shared molecular features associated with immune inflammation and metabolic dysregulation in gout and metabolic syndrome (MetS). We identified a set of common hub genes and observed in cellular models that JAK1 and CSF1R are associated with activation of the JAK–STAT pathway at the signaling level. We further suggest that the VISFATIN signaling pathway may represent a common axis linking these two diseases, with initial in vitro evidence supporting this association. Furthermore, we analyzed the potential roles of the ligand–receptor pairs NAMPT–(ITGA5 + ITGB1) in gout and NAMPT–INSR in MetS, highlighting their involvement in disease-related intercellular communication. Collectively, these findings, supported by bioinformatics and cellular experiments, provide insights into the intertwined pathophysiology of gout and MetS and may inform the identification of potential diagnostic biomarkers and therapeutic targets.

## Data Availability

The original contributions presented in the study are included in the article/Supplementary material, further inquiries can be directed to the corresponding author.
