# Supplementary figures and images for: Integrated multi-omics and single-cell transcriptomic analysis reveals shared molecular mechanisms and cell–cell communication signatures in gout and metabolic syndrome
Source: Front Med (Lausanne). 2026 Apr 15;13:1749788. doi: 10.3389/fmed.2026.1749788 (PMC13124607; doi:10.3389/fmed.2026.1749788)

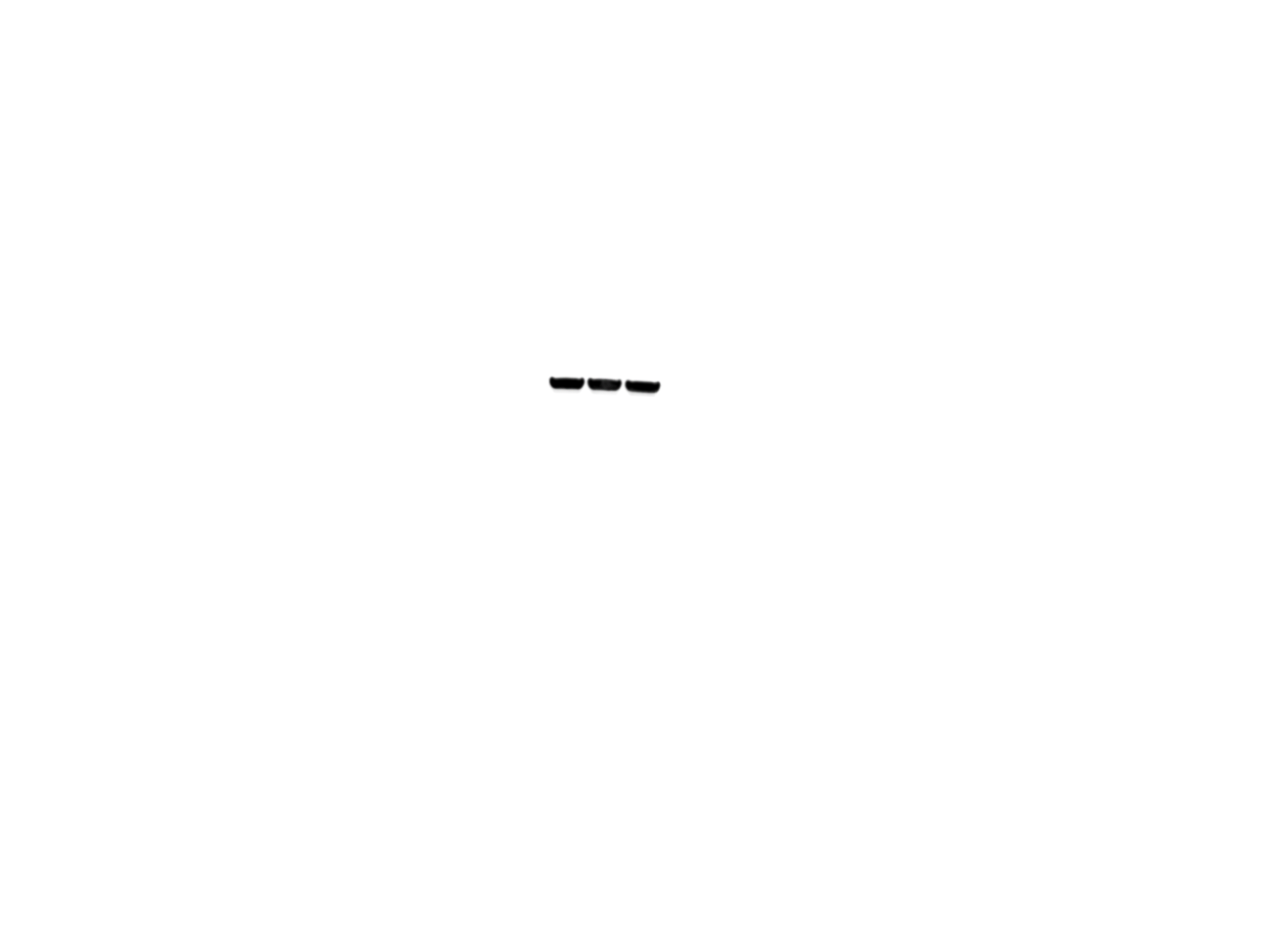

Supplement: Supplementary file 1 [file Data_Sheet_1.zip › WB╒√─ñ/ACTIN 42k.png]

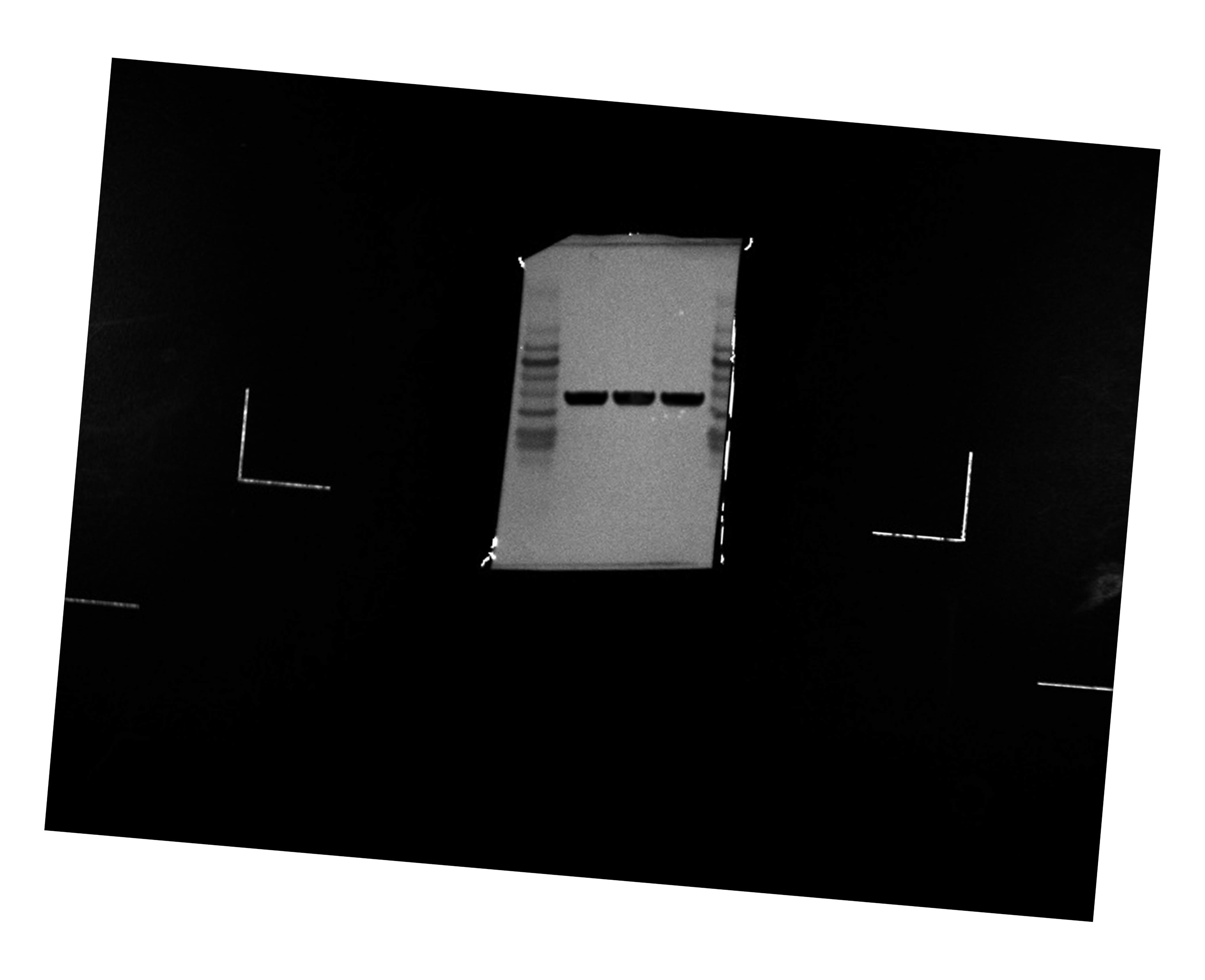

Supplement: Supplementary file 1 [file Data_Sheet_1.zip › WB╒√─ñ/ACTIN 42k░╫╣Γ.png]

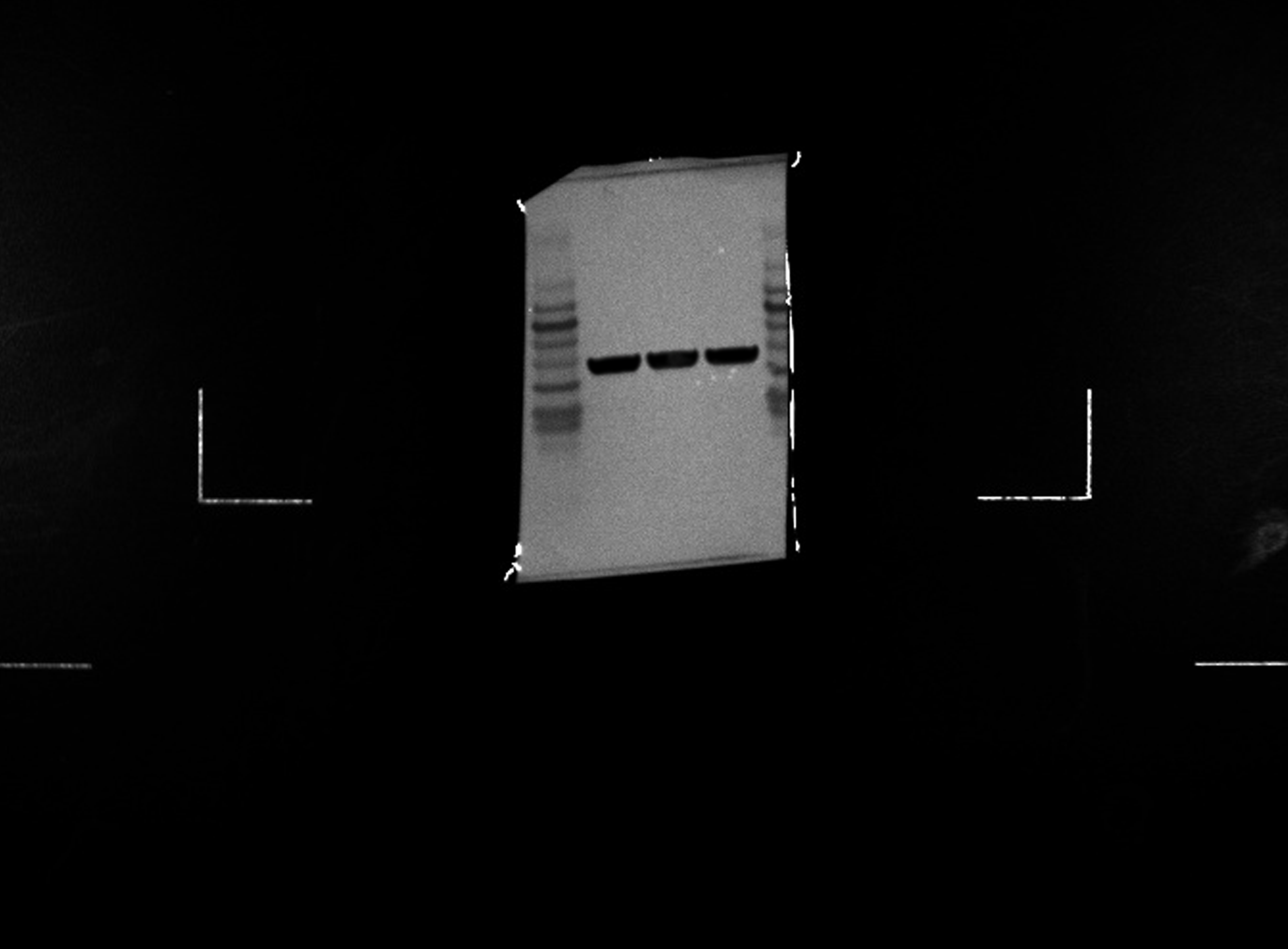

Supplement: Supplementary file 1 [file Data_Sheet_1.zip › WB╒√─ñ/ACTIN 42k░╫╣Γ1.png]

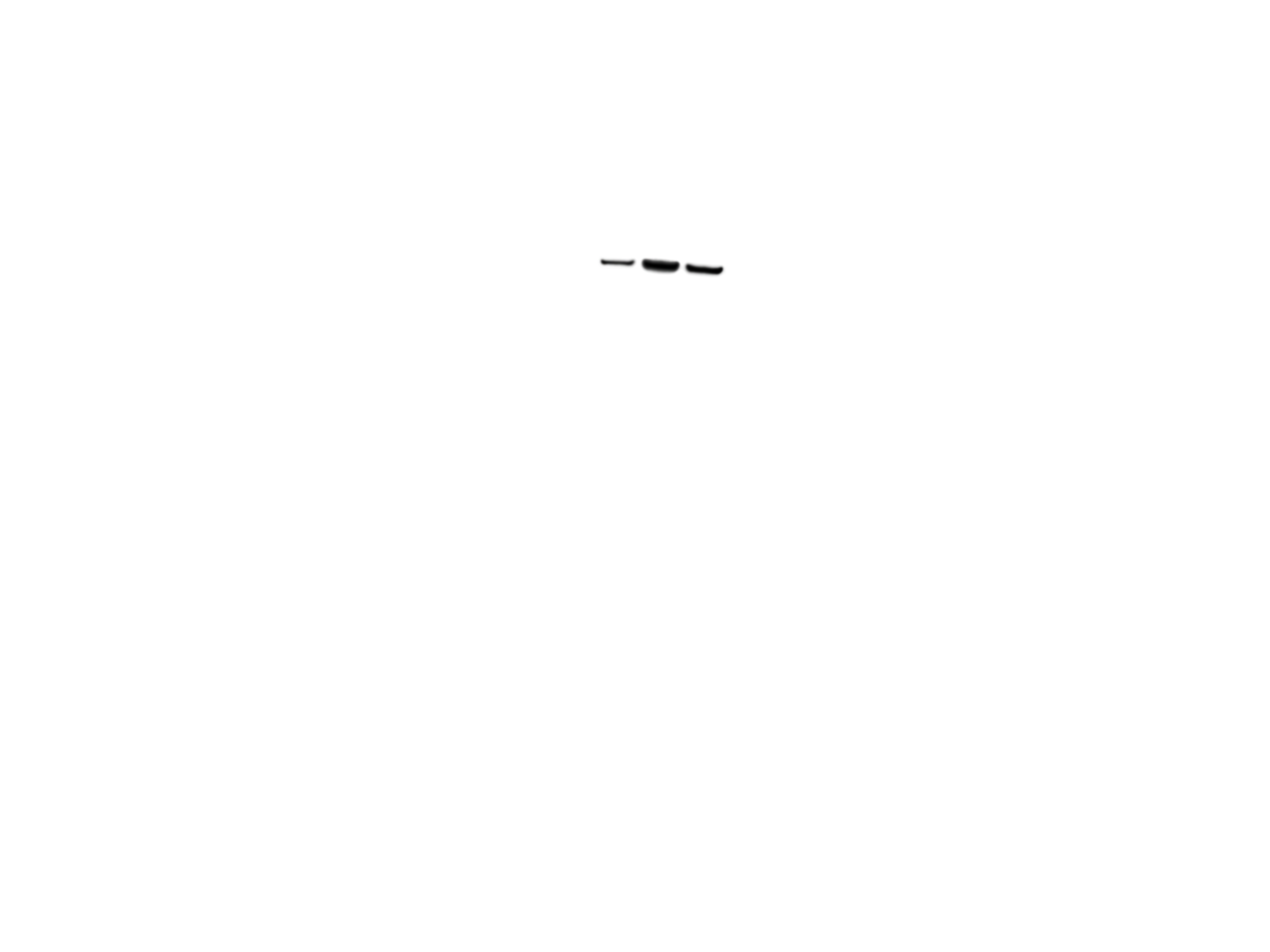

Supplement: Supplementary file 1 [file Data_Sheet_1.zip › WB╒√─ñ/CSF1R 108k.png]

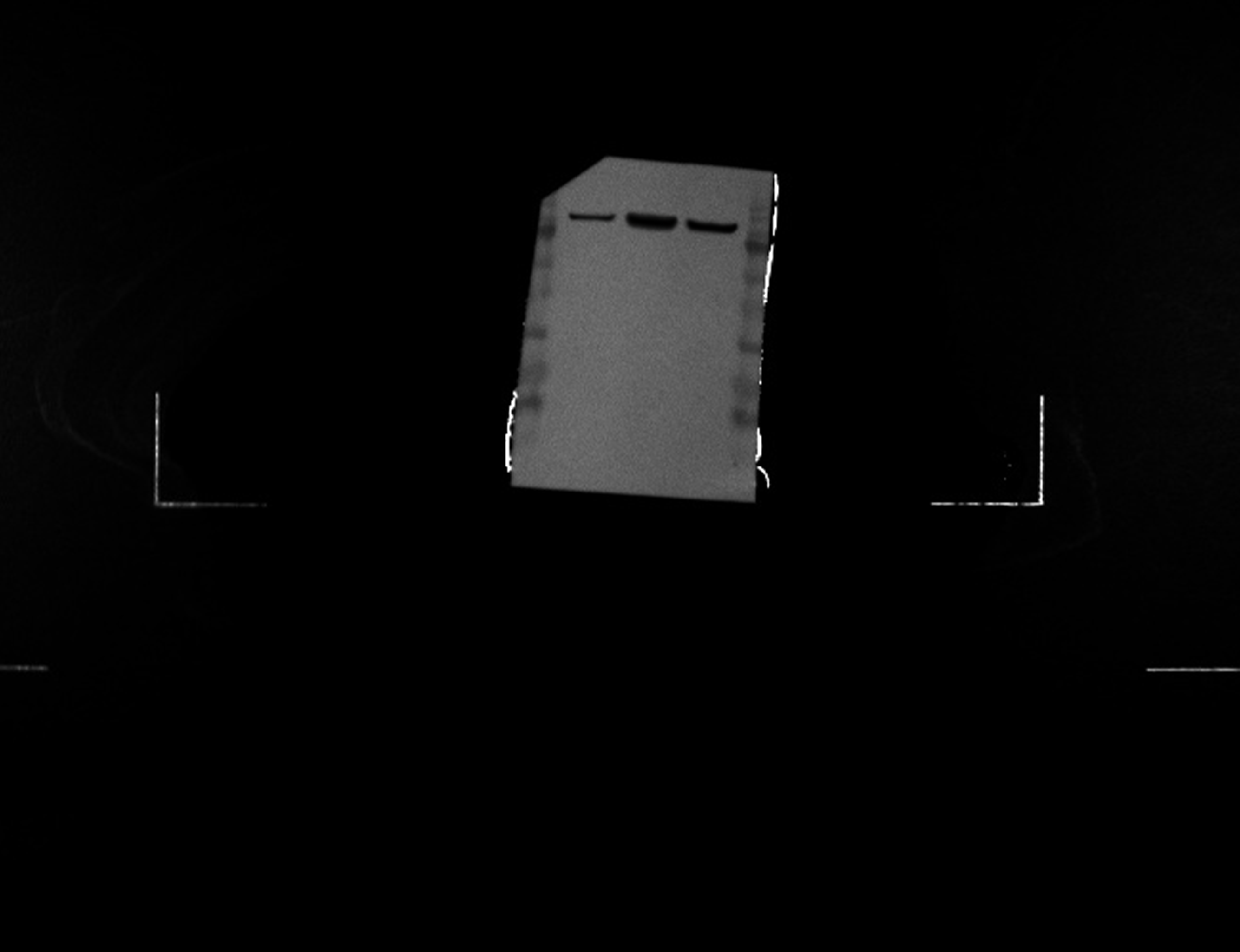

Supplement: Supplementary file 1 [file Data_Sheet_1.zip › WB╒√─ñ/CSF1R 108k░╫╣Γ.png]

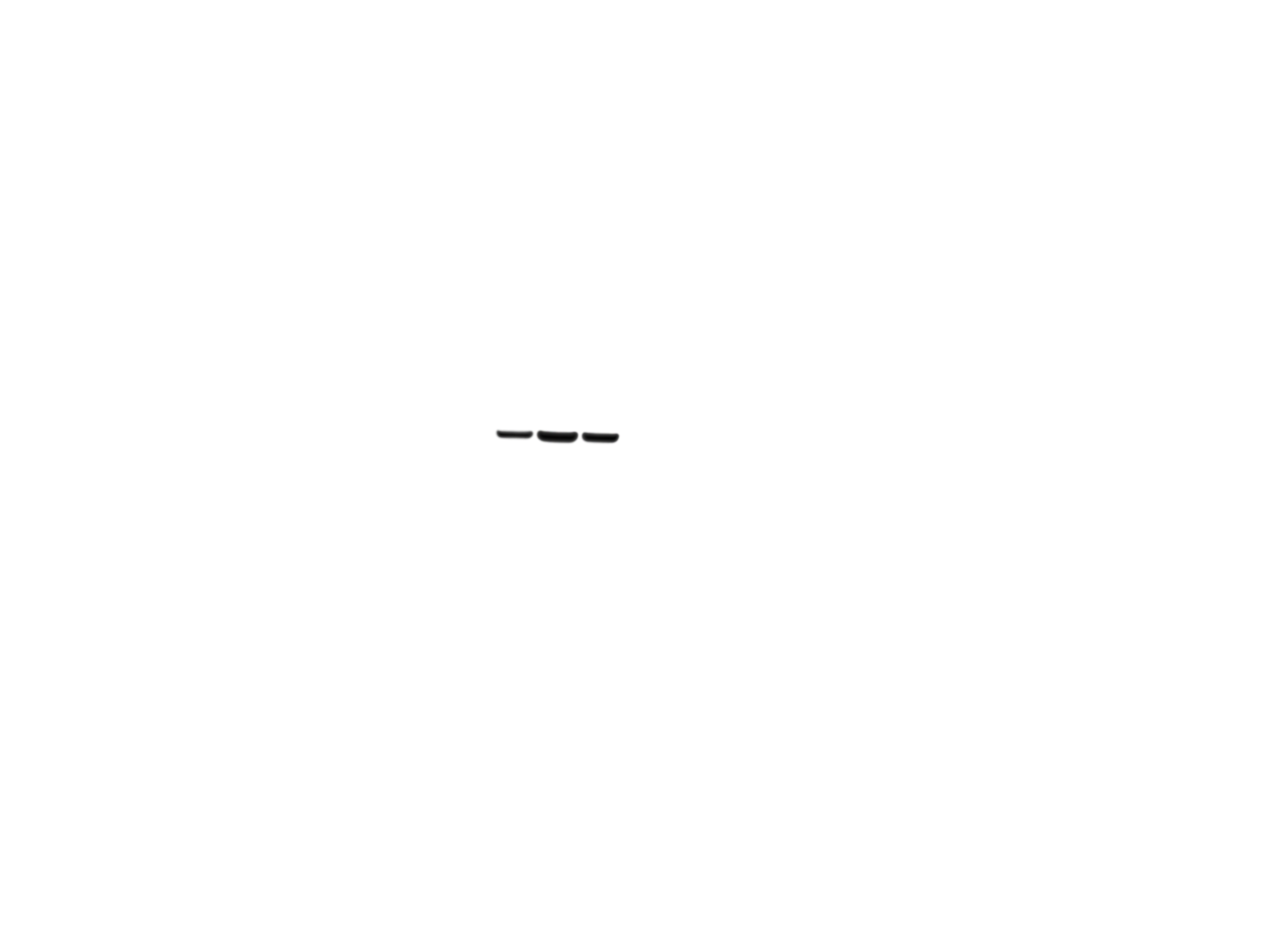

Supplement: Supplementary file 1 [file Data_Sheet_1.zip › WB╒√─ñ/JAK 134k.png]

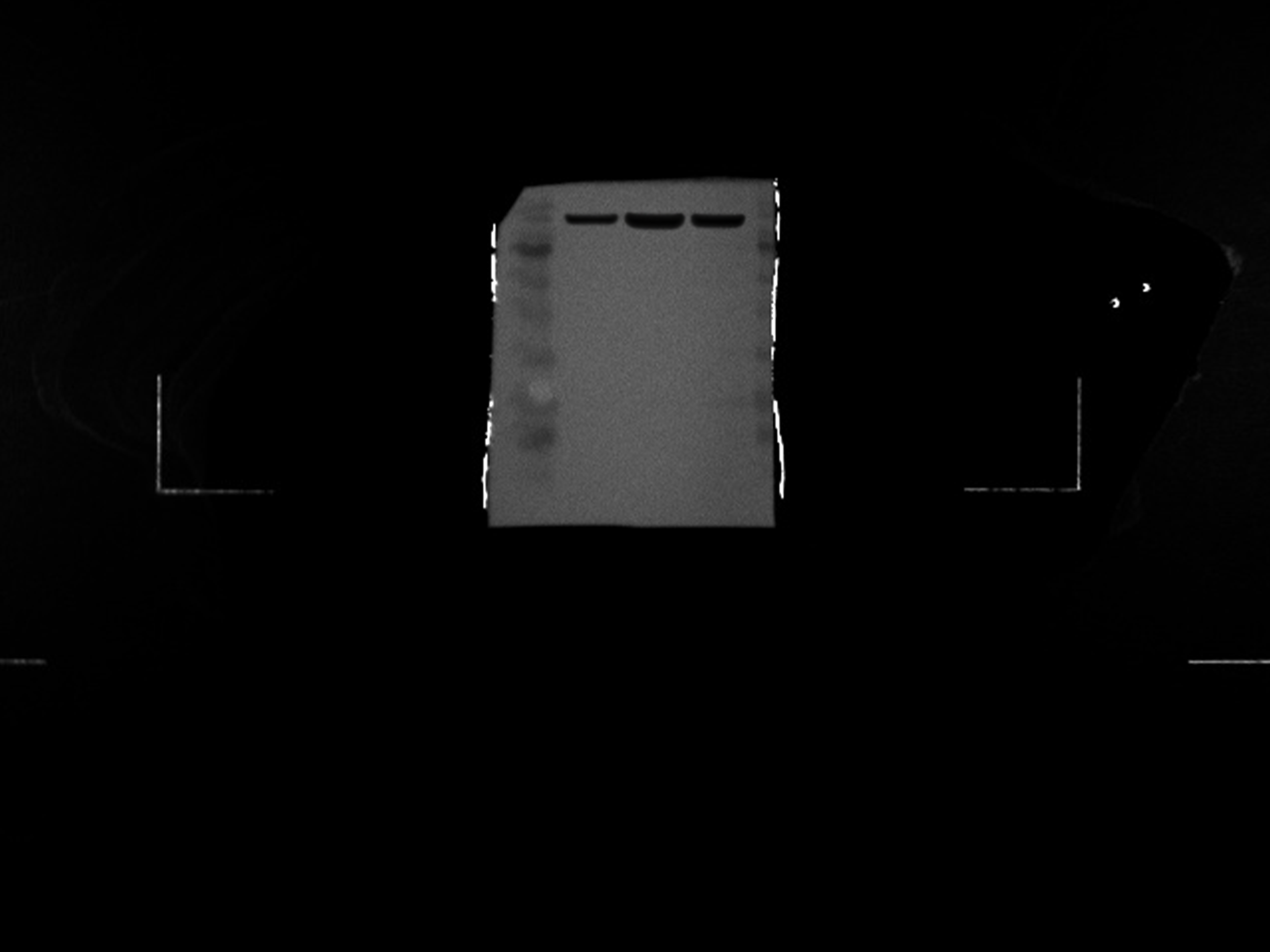

Supplement: Supplementary file 1 [file Data_Sheet_1.zip › WB╒√─ñ/JAK 134k░╫╣Γ.png]

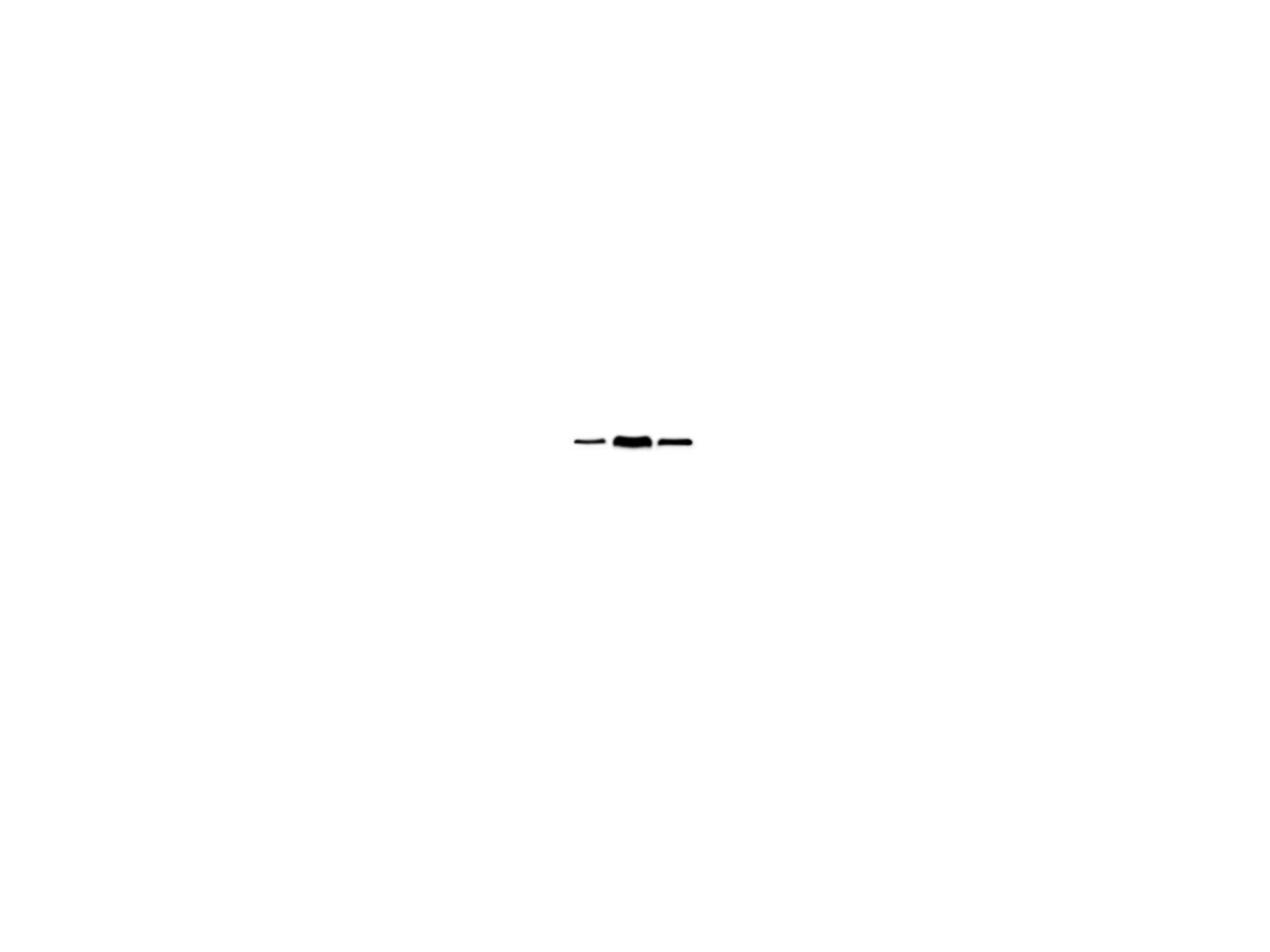

Supplement: Supplementary file 1 [file Data_Sheet_1.zip › WB╒√─ñ/P-STAT3 84k.png]

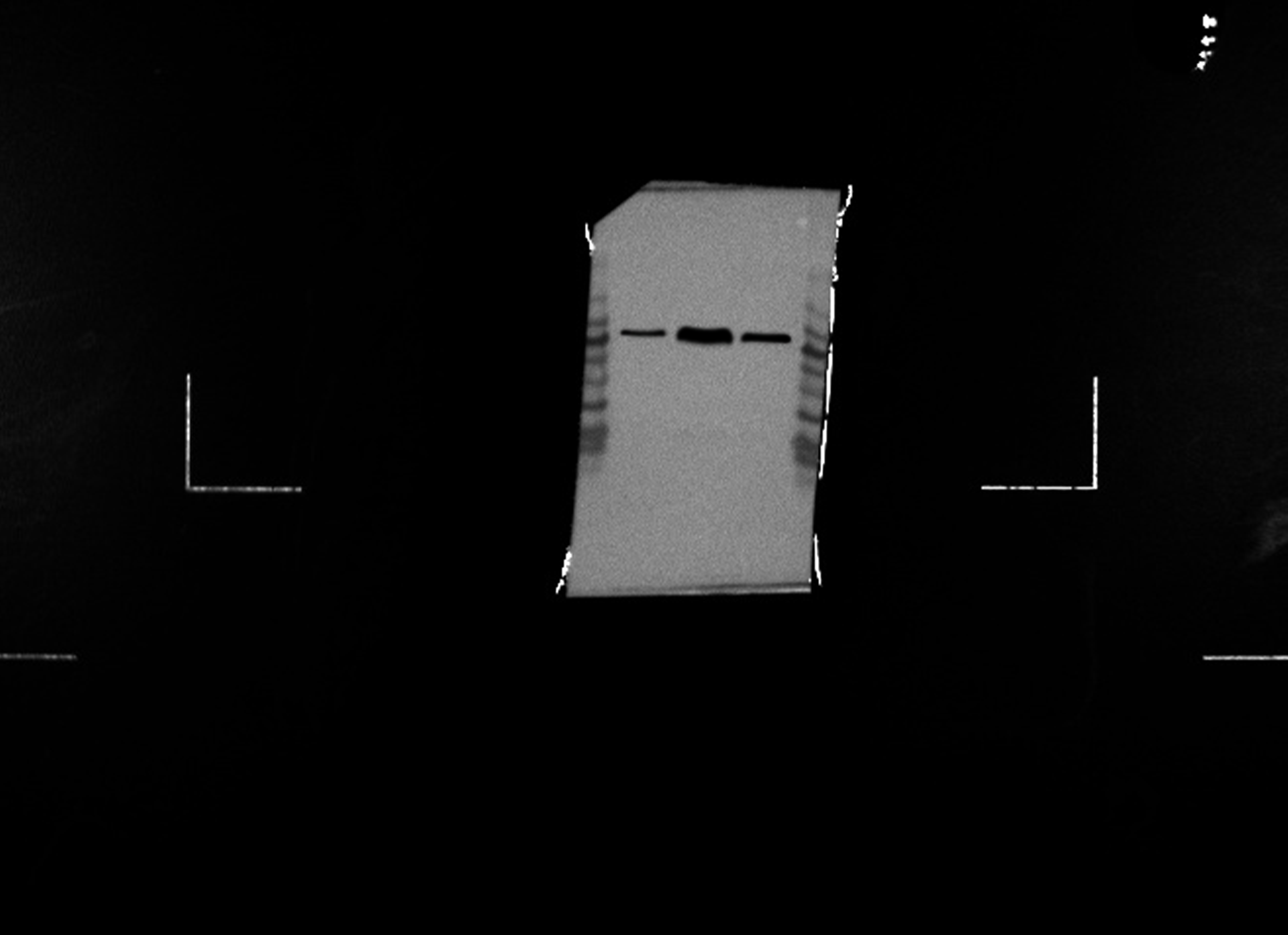

Supplement: Supplementary file 1 [file Data_Sheet_1.zip › WB╒√─ñ/P-STAT3 84k░╫╣Γ.png]
